# CRISPR diagnostics for maternal–infant HIV, hepatitis B, and syphilis care: clinical roles and translational gaps

**DOI:** 10.3389/fmicb.2026.1977927

**Published:** 2026-10-21

**Authors:** Hongzhi Luo, Lanke Lin, Fukang Luo, Xiaoxue Huang, Qiujie Yu, Qianyi Li, He Zha

**Affiliations:** 1 West China Hospital Sichuan University Jintang Hospital, Jintang First People’s Hospital, Chengdu, China; 2 Department of Laboratory Medicine, The Third Affiliated Hospital of Zunyi Medical University (The First People’s Hospital of Zunyi), Zunyi, Guizhou, China; 3 The Second Affiliated Hospital of Guizhou, University of Traditional Chinese Medicine, Guiyang, China; 4 Public Health Clinical Center of Chengdu, Chengdu, China

**Keywords:** CRISPR diagnostics, early infant diagnosis, hepatitis B virus, HIV, mother-to-child transmission, point-of-care testing, syphilis

## Abstract

This review asked which maternal–infant clinical decisions are plausible targets for CRISPR diagnostics, what evidence currently supports those roles, and what validation remains necessary. We conducted a structured narrative review of CRISPR diagnostics for HIV, hepatitis B virus (HBV), and syphilis, evaluating complete specimen-to-result systems rather than isolated nuclease reactions. Across the studies identified, HIV early infant diagnosis (EID) had the strongest near-term clinical rationale but lacked validation of microvolume whole blood or dried blood spots, broad HIV-1 inclusivity, and integrated process controls. Maternal HBV had the broadest clinical-specimen evidence, but no identified study established IU/mL-traceable classification around a pregnancy prophylaxis decision boundary. Syphilis direct detection supported a narrower specimen- and stage-dependent adjunctive role and could not replace antenatal serology or exclude congenital infection when negative. Major cross-cutting gaps included specimen preparation, calibration, contamination containment, invalid-result detection, and multisite intended-use validation. These findings do not establish routine clinical readiness. As an evidence-informed development priority, a shared service platform with separately validated pathogen-specific assays appears more defensible in the near term than a universal single-tube triplex.

## 1 Introduction

Mother-to-child transmission of HIV, HBV, and syphilis remains an important but largely preventable cause of fetal, neonatal, and infant morbidity and mortality. The three infections differ in when transmission occurs and in the interventions required to prevent it. HIV can be transmitted during pregnancy, labor, delivery, or breastfeeding; prevention therefore relies on early maternal diagnosis, sustained antiretroviral therapy (ART) and viral suppression, appropriate peripartum and neonatal prophylaxis, and timely early infant diagnosis (World Health Organization, 2022, 2024b, 2025). HBV transmission occurs mainly around delivery and is strongly associated with maternal viremia. Antenatal hepatitis B surface antigen (HBsAg) screening, assessment for maternal antiviral prophylaxis, and prompt infant vaccination, including the birth dose, form a linked prevention pathway (Pan et al., 2016; Jourdain et al., 2018; World Health Organization, 2020, 2024a). *Treponema pallidum (T. pallidum)* can cross the placenta during pregnancy; without adequate maternal treatment, infection may lead to fetal loss, prematurity, congenital infection, or neonatal disease (World Health Organization, 2016, 2017).

Despite these biological differences, prevention is delivered through overlapping antenatal, delivery, immunization, and child-health services. The WHO triple-elimination framework uses this shared service platform to reduce repeated visits and missed opportunities. Its success depends not simply on performing more tests, but on returning pathogen-appropriate results early enough to trigger effective maternal and infant interventions (Zhang et al., 2019; World Health Organization, 2022, 2024b, 2025).

Established serological and antigen-based algorithms already address most initial antenatal screening needs across HIV, HBV, and syphilis (Selph et al., 2019; Henderson et al., 2019; Lin et al., 2018). Molecular testing is therefore most defensible when it answers a specific downstream decision that screening alone cannot resolve. Maternal HIV care requires calibrated viral-load measurement, whereas HIV EID requires highly sensitive nucleic acid testing because maternally acquired antibodies limit early serology (Wesolowski et al., 2020; Ochodo et al., 2021). After HBsAg positivity, the key HBV question is whether maternal viremia meets locally applicable criteria for antiviral prophylaxis or whether an accepted surrogate pathway applies (Boucheron et al., 2021; World Health Organization, 2024a). For syphilis, direct detection has a narrower role, principally when a compatible lesion, a possible seronegative window, or discordant clinical and serological findings justify additional pathogen evidence (Vrbová et al., 2020; Theel et al., 2020). A shared reader or clinic workflow may therefore still require different specimens, cartridges, calibration rules, and interpretive algorithms.

The diagnostic challenge is also organizational. Late entry to antenatal care, referral of reactive specimens, delayed result return, incomplete delivery records, and loss to infant follow-up can interrupt an otherwise effective prevention pathway. Near-patient testing may reduce these gaps, but rapid signal generation alone does not ensure clinical impact. Experience with conventional point-of-care EID shows that shorter turnaround can accelerate ART initiation, while retention, stock management, operator behavior, and referral systems remain decisive (Finocchario-Kessler et al., 2018; Jani et al., 2018; Mwenda et al., 2018). New diagnostics should therefore be judged by their ability to function within the complete care pathway, not only by analytical performance in controlled laboratory conditions.

Clustered regularly interspaced short palindromic repeats diagnostics use RNA-guided nucleases, most commonly Cas12 or Cas13, to recognize pathogen-specific nucleic acid sequences, and their broader diagnostic potential has been reviewed across multiple assay formats (Gootenberg et al., 2017, 2018; Chen et al., 2018; Ackerman et al., 2020; Kaminski et al., 2021; Mohammadhassan et al., 2022). Most diagnostic formats pair CRISPR recognition with PCR or isothermal amplification and a fluorescent, colorimetric, or lateral-flow readout. For maternal–infant use, however, clinical performance depends on the complete specimen-to-result system–not nuclease chemistry alone–including target recovery, effective specimen volume, sequence inclusivity, calibration where required, contamination control, and detection of invalid results. Reaction-level limits of detection or a portable readout therefore do not establish clinical readiness. The specimen-to-decision framework used throughout this review is summarized in Figure 1.

**FIGURE 1:**
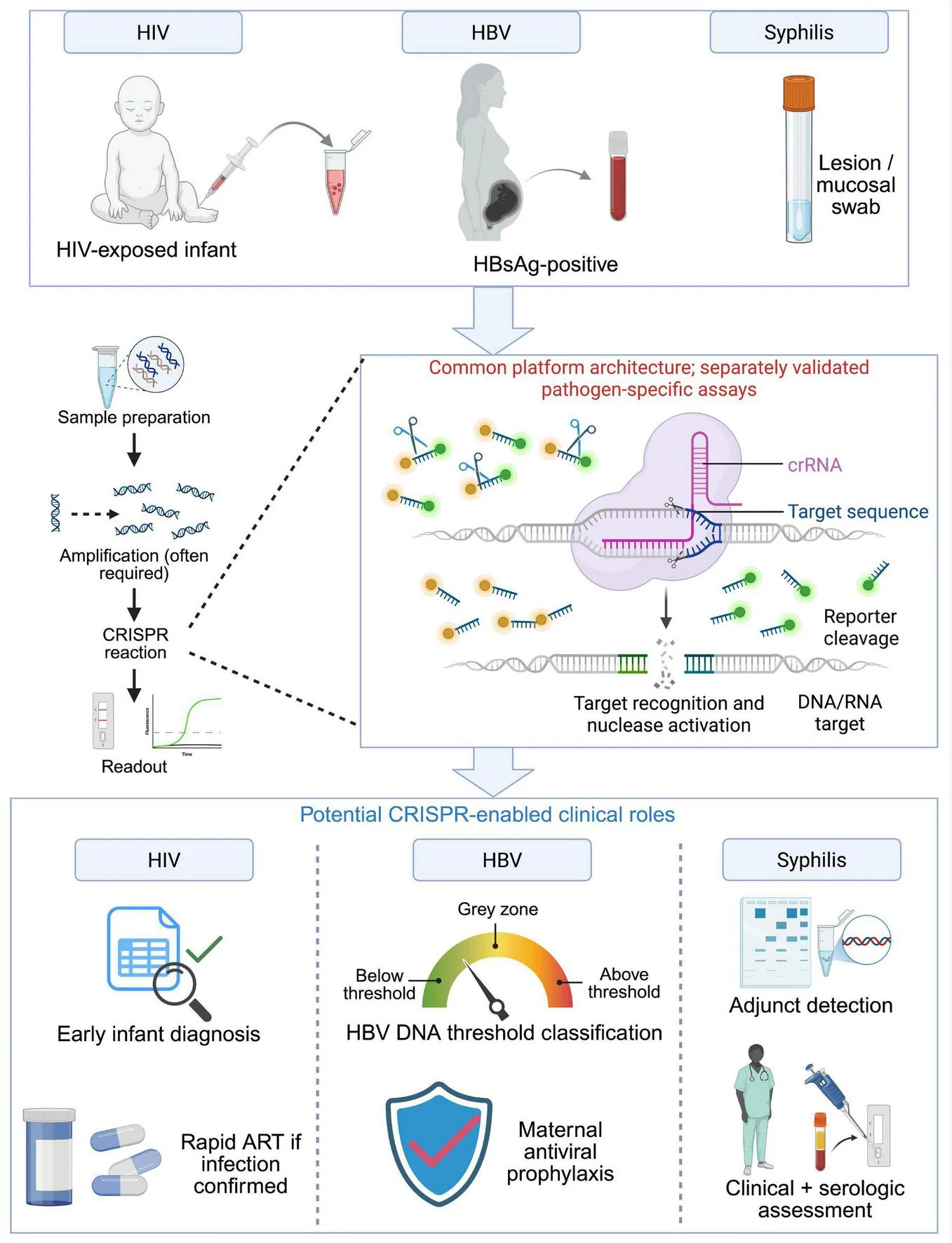
Conceptual specimen-to-decision architecture of CRISPR diagnostics for maternal–infant HIV, HBV, and syphilis care. Representative pathogen-specific specimens and clinical applications are linked through a representative Cas12/Cas13-type workflow comprising sample preparation, amplification where required, CRISPR-mediated target recognition and nuclease activation, and signal readout. The illustrated outputs correspond to qualitative HIV early infant diagnosis (EID), HBV DNA threshold classification to inform maternal antiviral prophylaxis, and adjunctive *Treponema pallidum* detection interpreted with clinical and serological assessment. The schematic represents a shared platform concept with separately validated pathogen-specific assays rather than a universal triplex test. ART, antiretroviral therapy; CRISPR, clustered regularly interspaced short palindromic repeats; crRNA, CRISPR RNA; EID, early infant diagnosis; HBsAg, hepatitis B surface antigen; HBV, hepatitis B virus; HIV, human immunodeficiency virus.

Previous reviews have commonly organized CRISPR diagnostics by nuclease, amplification chemistry, or readout modality (Kaminski et al., 2021). Here, the maternal–infant clinical pathway provides the organizing framework. We examine which clinical decisions are plausible targets for CRISPR testing, what specimen and assay requirements follow from those decisions, and what evidence supports HIV EID, maternal HBV reflex classification, and adjunctive *T. pallidum* detection. A central systems-level question addressed by this review is whether triple-elimination services require a universal triplex chemistry or may be better served by shared service infrastructure with separately validated pathogen-specific assays. We therefore compare reaction-level multiplexing, cartridge-level multiplexing, and service-platform integration in relation to the distinct analytes, specimens, outputs, and clinical actions required for HIV, HBV, and syphilis. By linking analytical claims to clinical actions and consequences of error, this review defines the narrowest defensible roles for current systems and the evidence needed for translation.

### 1.1 Literature search and study selection

We conducted a structured narrative review organized around the maternal–infant clinical pathway. A structured narrative design was selected because the review question concerns clinical positioning across heterogeneous assay architectures, specimen types, outputs, and evidence stages rather than estimation of a single pooled diagnostic effect. Structured searching and explicit eligibility, extraction, and appraisal procedures were used to improve transparency and reproducibility without implying systematic-review exhaustiveness. PubMed/MEDLINE and Europe PMC were searched from database inception to 18 August 2026 for original CRISPR/Cas diagnostic studies targeting HIV, HBV, or *T. pallidum*, supplemented by citation chaining and targeted retrieval of relevant guidance and comparator evidence. Core pathogen-specific studies were classified by evidence stage to describe study maturity and appraised using an author-defined five-domain framework to support clinical positioning: direct intended-use evidence, intended-specimen integration, measurement and interpretation validity, control and containment maturity, and operational or clinical-utility evidence. Evidence stage and diagnostic-accuracy design were treated as distinct attributes: evidence stage described study maturity, whereas QUADAS-2 was used only when a patient-specimen study addressed a diagnostic-accuracy question with an applicable reference standard (Whiting et al., 2011). The five-domain framework was not used as a pass/fail checklist; each study was interpreted against its stated analytical, engineering, or clinical claim.

Owing to substantial clinical and analytical heterogeneity, findings were synthesized narratively rather than meta-analyzed. The review was not prospectively registered as a systematic review, and retrospective PRISMA-style screening counts were not reconstructed. The review workflow is summarized descriptively in Supplementary Figure 1; full search strategies, eligibility and screening procedures, data-extraction fields, and appraisal methods are provided in Supplementary Table 1, with study-level evidence and QUADAS-2 judgments reported in Supplementary Tables 2, 3.

## 2 Clinical decision points and unmet diagnostic needs across the maternal–infant care continuum

### 2.1 Clinically actionable windows across pregnancy, delivery, and infancy

At the earliest feasible antenatal encounter, the objective is timely identification of women who require treatment, further testing, or intensified prevention rather than the generation of a complex molecular profile (Selph et al., 2019). Mature serological or antigen-based pathways already serve most maternal HIV, HBV, and syphilis screening needs (Lin et al., 2018; Henderson et al., 2019). Replacing them with nucleic acid CRISPR assays would require evidence of improved coverage, turnaround, affordability, or linkage to care without loss of established diagnostic performance (Land et al., 2018).

A molecular CRISPR test could be considered when conventional screening is genuinely inaccessible, when a clinically important preseroconversion window exists, or when molecular testing infrastructure is already available for other indications. Nevertheless, universal molecular screening would impose target-recovery requirements that are often more demanding than antigen or antibody detection. The relevant comparator is therefore not an idealized central-laboratory PCR alone; it is the entire existing testing algorithm, including rapid tests that may already return actionable results during the same visit (Lee et al., 2012; Ndlovu et al., 2018).

After a reactive screen, the clinical rationale for molecular reflex testing becomes stronger because a smaller population requires confirmation, quantification, or risk classification. HIV confirmatory testing and maternal viral load measurement have distinct purposes (Wesolowski et al., 2020; Chilaka and Konje, 2021). For HBV, a positive HBsAg result should trigger assessment of viral replication or an accepted surrogate when this information guides prophylaxis. WHO guidance supports improved access to point-of-care HBV DNA testing and reflex approaches while retaining HBsAg screening (World Health Organization, 2024a). In syphilis, discrepant serology, previous treatment, or possible early infection may require repeat serology, clinical adjudication, or selected direct detection rather than a universal molecular reflex (Mmeje et al., 2015; Vrbová et al., 2020).

Reflex testing has greatest clinical value when it prevents a second visit, reduces loss to follow-up, or accelerates an intervention. A CRISPR cartridge that delivers HBV DNA threshold classification at the antenatal clinic could therefore have greater impact than a more sensitive qualitative HBV assay performed in a central laboratory requiring specimen referral (Hajarizadeh et al., 2026). The same principle applies to HIV viral-load testing: a decentralized assay must produce a sufficiently calibrated result to support management, not merely detect residual RNA (Sacks et al., 2019).

At delivery and immediately after birth, tolerance for delay is minimal. Missing maternal records may justify rapid testing, but testing must not postpone interventions that should proceed on clinical or guideline grounds (Byrne et al., 2025). HBV nucleic acid testing may refine maternal risk classification, yet it cannot replace a timely vaccine birth dose or other indicated immunoprophylaxis. HIV results must be linked immediately to the relevant peripartum and neonatal antiretroviral pathway. For syphilis, the timing and adequacy of maternal penicillin treatment, infant examination, serology, and follow-up often carry more weight than a single direct-pathogen result. A delivery-room assay should therefore minimize operator-dependent preparation, identify inadequate specimens and process failures, and remain stable under realistic workload and environmental conditions. Rapid signal generation alone is not sufficient (Spooner et al., 2019; Tsai et al., 2019; Fanella et al., 2024).

During infancy, HIV EID represents the clearest need for molecular testing because maternal antibody persists and treatment of infected infants is urgent (Violari et al., 2008). The assay must accommodate heel-prick capillary whole blood, low-volume venous blood, or dried blood spots and retain sensitivity when infant prophylaxis reduces viral abundance. A negative result at one time point does not necessarily complete the testing algorithm; age, exposure timing, breastfeeding, and prophylaxis determine the need for repeat testing (Patton et al., 2007; Balasubramanian et al., 2017).

Hepatitis B virus infant follow-up has a different purpose. Infection status and vaccine-induced protection are not interchangeable outcomes. HBsAg, antibody to hepatitis B surface antigen (anti-HBs), and nucleic acid tests provide different information, and a CRISPR nucleic acid result cannot assess protective antibody (World Health Organization, 2024a; Schillie et al., 2015). For congenital syphilis, no single laboratory result can replace integrated assessment of maternal treatment history, paired serology where appropriate, clinical findings, and follow-up (World Health Organization, 2016, 2017). These distinctions require each pathogen-specific endpoint to be validated separately. The corresponding decision points across pregnancy, delivery, the neonatal period, and infancy are mapped in Figure 2.

**FIGURE 2:**
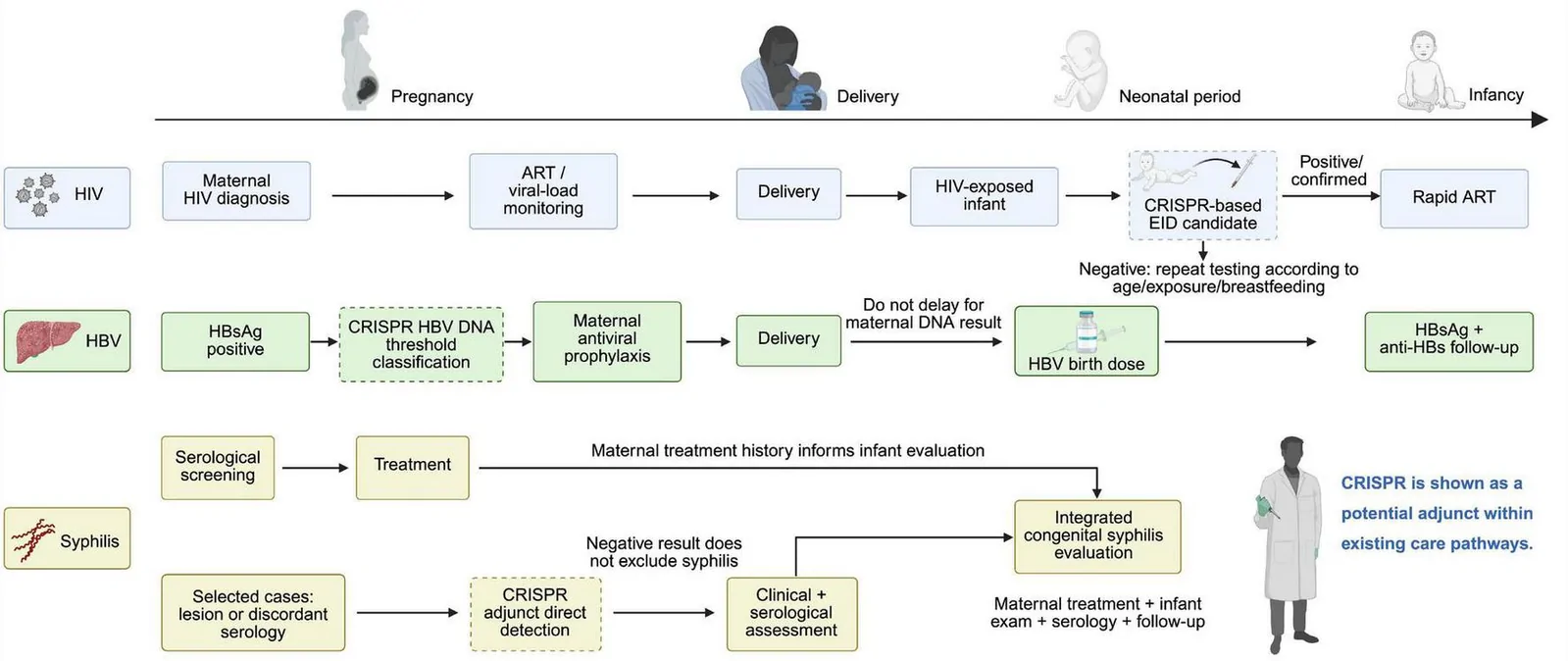
Pathogen-specific diagnostic decision points across the maternal–infant care continuum. The schematic maps pregnancy, delivery, the neonatal period, and infancy and illustrates potential positions for CRISPR testing within established HIV, HBV, and syphilis care pathways. CRISPR is positioned as a candidate approach for HIV early infant diagnosis, HBV DNA threshold classification after maternal HBsAg positivity, and adjunctive direct detection of *Treponema pallidum* in selected cases. Established preventive, treatment, serological, and follow-up pathways remain central to clinical management. anti-HBs, antibody to hepatitis B surface antigen; ART, antiretroviral therapy; CRISPR, clustered regularly interspaced short palindromic repeats; EID, early infant diagnosis; HBsAg, hepatitis B surface antigen; HBV, hepatitis B virus; HIV, human immunodeficiency virus.

### 2.2 HIV: maternal viral-load assessment and early infant diagnosis

Maternal HIV viral load monitoring requires quantitative results with a documented calibration hierarchy and clinically acceptable agreement with a reference method (Davis et al., 2003). For a narrowly defined urgent decision, a validated threshold classifier may be sufficient, but its intended action and uncertainty zone must be prespecified (Padoan et al., 2018). Relevant characteristics include a broad dynamic range, precision across runs and sites, reliable handling of high concentrations without saturation, and sensitivity at low concentrations in treated women (Swenson et al., 2014). Inclusivity across HIV-1 subtypes and circulating recombinant forms is essential because mismatches in either the crRNA or amplification primers can reduce signal (Molina Vargas et al., 2024). A qualitative “HIV detected” result is not equivalent to viral load monitoring. Unlike the proposed HBV prophylaxis classifier, maternal HIV management often requires longitudinal interpretation of suppression, rebound, and change over time; a binary threshold test would therefore have a narrower triage role and would not generally replace quantitative monitoring (Sha et al., 2011).

For HIV-exposed infants, EID requires a target product profile distinct from maternal viral-load monitoring. The priority is clinically sensitive qualitative detection with a valid result from the specimen and volume intended for use. A credible assay should recover low-copy HIV nucleic acid from microvolume whole blood or dried blood spots, remain inclusive across relevant HIV-1 subtypes and recombinants, and retain performance when prophylaxis suppresses viral abundance. Specimen-adequacy and full-process controls should identify poor collection, extraction failure, inhibition, and reagent failure. Prospective studies should report false-negative results by reference viral-load stratum and prophylaxis exposure, together with invalid-test, repeat-test, and time-to-treatment outcomes. For EID, clinically informative validation should therefore emphasize low-positive performance in the intended specimen, invalid and repeat rates, prophylaxis exposure, and time to treatment (Finocchario-Kessler et al., 2018; Jani et al., 2018; Mwenda et al., 2018).

### 2.3 HBV: screening, maternal risk stratification, and infant follow-up

Because HBsAg remains the established first-line marker for maternal HBV screening, CRISPR is more plausibly positioned as rapid HBV DNA reflex testing after a positive screen. Such testing could avoid specimen referral or a return visit and, where prophylaxis eligibility is linked to viremia, provide an actionable result during the same encounter. Under the 2024 WHO guidance, when HBV DNA or hepatitis B e antigen (HBeAg) testing is available, tenofovir disoproxil fumarate prophylaxis is recommended for HBsAg-positive pregnant women with HBV DNA ≥ 200,000 IU/mL or positive HBeAg; when neither HBV DNA nor HBeAg testing is available, prophylaxis for all HBsAg-positive pregnant women may be considered according to programme context (World Health Organization, 2024a). These interventions are additional to timely infant hepatitis B vaccination, including the birth dose. The diagnostic task for a molecular reflex assay is therefore not simply to detect HBV DNA, but to classify maternal viremia reliably in relation to the locally applicable action rule.

The value of HBV DNA threshold classification is programme dependent. It is greatest where antiviral eligibility is explicitly linked to HBV DNA and laboratory referral causes clinically important delay. Where a validated HBeAg pathway or broader prophylaxis strategy is used, the incremental value of decentralized HBV DNA testing may shift from eligibility determination to confirmation, monitoring, or programme quality assurance (World Health Organization, 2024a; Delamare et al., 2024; Ndow et al., 2025). Target product profiles should therefore be adaptable to national guidance rather than assume a single immutable decision rule.

A threshold classifier may be simpler than a full viral load analyzer, but it still requires rigorous validation. Validation cohorts should deliberately oversample specimens near the decision boundary. Replicate measurements should define imprecision, and a prespecified gray zone should trigger repeat or laboratory testing. Results should be calibrated against a reference method traceable to the WHO International Standard rather than inferred from plasmid copies without commutability evidence. High-load specimens require assessment for signal saturation, whereas lower concentrations require evaluation of stochastic sampling and extraction loss (Coste and Pouchot, 2003; Forootan et al., 2017).

Rapid maternal HBV DNA testing at delivery could be useful for women with late or absent antenatal care, but it must not delay the infant hepatitis B birth dose. WHO recommends administering the hepatitis B vaccine birth dose as soon as possible after birth, preferably within 24 h, as part of the universal infant schedule; additional immunoprophylaxis may be indicated according to national policy and maternal risk (World Health Organization, 2020, 2024a). The value of a CRISPR result would lie in supporting urgent maternal management and linkage to appropriate infant prophylaxis, not in replacing immediate newborn action.

Interpretation of HBV DNA detected at birth depends on sampling time, specimen type, and the longitudinal follow-up algorithm. A single birth-time result does not establish persistent infant infection. Later HBsAg and anti-HBs testing evaluates infection and immunization outcomes that DNA testing alone cannot capture. CRISPR may contribute as a molecular adjunct where standard nucleic acid testing is unavailable, but it should not replace the recommended longitudinal follow-up algorithm (Chen et al., 2013; Schillie et al., 2015).

Development of HBV assays should prioritize rapid reflex access after HBsAg positivity and threshold classification based on results traceable to international units. Performance must remain stable near the intervention threshold and across relevant genotypes in a decentralized workflow. The analytical advantages of relatively high viremia and DNA stability do not compensate for poor threshold classification (Maasoumy et al., 2017).

### 2.4 Syphilis: serological screening and selective direct detection

The central public-health objective in pregnancy is timely diagnosis followed by adequate penicillin treatment. WHO recommendations therefore remain centered on serological screening and treatment rather than routine pathogen nucleic acid testing (World Health Organization, 2016, 2017). Persistent treponemal reactivity after previous infection and the need to interpret non-treponemal titers can create difficult cases, but these limitations do not justify replacing rapid serology. A CRISPR assay detecting *T. pallidum* DNA is better positioned as complementary evidence when a compatible lesion is present during a possible seronegative window, when serological results are discordant, or when reinfection is suspected and an appropriate lesion or mucosal specimen can be collected. Placental, cord, tissue, or neonatal specimens require separate validation and should remain investigational rather than being extrapolated from lesion or blood studies.

Clinical performance must be reported by specimen and stage (Tipple et al., 2011). Lesion exudate or swabs in primary and secondary syphilis may contain substantially higher organism burdens than blood. Whole blood, serum, or plasma may contain low organism burdens; a negative result may represent stochastic absence in the aliquot rather than absence of infection. Placenta, cord, tissue, amniotic fluid, and cerebrospinal fluid pose different issues of sampling, contamination, invasiveness, and reference-standard uncertainty. Combining these matrices into one sensitivity estimate obscures matrix-dependent determinants of test performance (Grange et al., 2012; Gayet-Ageron et al., 2013; Ryan et al., 2026).

Disease stage is as important as specimen type (Gayet-Ageron et al., 2013; Pinto et al., 2017). Direct detection is most plausible in organism-rich early disease and becomes less reliable after lesions resolve or when organisms are sparse. Treatment may reduce viable pathogen while residual nucleic acid persists for an uncertain period; a positive result after treatment therefore does not by itself indicate viability, treatment failure, or ongoing transmissibility. In congenital syphilis, maternal treatment history, infant findings, paired serology, and longitudinal follow-up remain central, and a negative CRISPR result cannot exclude infection. Each intended specimen should consequently have its own adequacy and full-process controls, because both target non-detection and persistent target detection can be misleading.

Table 1 summarizes the pathogen-specific clinical decisions, intended specimens and outputs, and principal consequences of error that frame the evidence appraisal in subsequent sections. These clinical decision points define the intended-use benchmarks against which the pathogen-specific evidence in Section “4 Pathogen-specific evidence and translational readiness” is appraised; cross-cutting assay-design requirements are addressed once in Section “3 Design requirements for maternal and infant CRISPR testing.”

**TABLE 1:** Established or guideline-based diagnostic pathways, pathogen-specific clinical decisions, required outputs, and principal consequences of error.

Clinical application	Established/guideline pathway and clinical decision	Intended analyte/specimen	Required output	Principal error consequence	CRISPR incremental value/validation emphasis
Maternal HIV viral load	Assess suppression, rebound, or urgent peripartum risk; comparator: calibrated quantitative NAT.	HIV RNA in plasma or a separately validated whole-blood workflow.	Traceable quantitative result; threshold output only for a prespecified action.	Bias or imprecision may misclassify management or obscure longitudinal change.	Shorter actionable turnaround without loss of agreement, range, subtype inclusivity, or error control; near-term role: investigational triage.
HIV EID	Diagnose infection in an HIV-exposed infant and enable rapid ART; comparator: validated qualitative NAT.	HIV RNA, proviral DNA, or total nucleic acid in microvolume whole blood, dried blood spots, or plasma; analyte-specific claims.	Positive, negative, invalid, or indeterminate qualitative result.	Low-load false negatives delay ART; false positives cause unnecessary ART and distress.	Low-target performance in prophylaxis-exposed infants; robust recovery, full-process controls, subtype coverage, and shorter time to ART.
Maternal HBV reflex testing	Classify prophylaxis eligibility after HBsAg positivity; comparator: laboratory HBV DNA or a validated surrogate pathway.	HBV DNA in whole blood, plasma, or serum.	IU/mL-linked threshold classification with prespecified gray-zone and out-of-range rules.	Threshold error may deny or unnecessarily trigger prophylaxis.	Same-visit value; low boundary error across genotypes, lots/operators, and high loads; traceability to IU/mL.
HBV infant follow-up	Assess infection and immunization outcomes at guideline-defined time points; comparator: HBsAg/anti-HBs, with molecular testing when indicated.	HBV DNA plus established serological markers.	Longitudinal interpretation; DNA does not replace HBsAg/anti-HBs.	Birth-time DNA may be overinterpreted as persistent infection or protection.	Molecular adjunct only; validate sampling time and integrate with established serological follow-up.
Syphilis adjunct direct detection	Add pathogen evidence in early/discordant or suspected reinfection cases; comparator: treponemal/non-treponemal serology plus clinical assessment.	*T. pallidum* DNA; lesion or mucosal swab preferred for an initial claim.	Qualitative, specimen- and stage-specific result.	Negative direct tests may falsely reassure; post-treatment positives may be mistaken for viable infection.	Show incremental value beyond serology; validate each specimen/stage separately; negative results require a non-exclusion qualifier.
Congenital syphilis evaluation	Support case classification and management; comparator: maternal treatment history, infant examination, serology, and follow-up.	Placental/other tissue, lesion material, cord blood/tissue, or neonatal blood; each requires separate validation.	Supplementary evidence only; not a stand-alone exclusion result.	False reassurance or overinterpretation may distort infant treatment and follow-up.	Investigational; use integrated clinical adjudication and do not pool matrices into one sensitivity estimate.

The incremental values listed here represent requirements for comparative validation and should not be interpreted as demonstrated advantages of CRISPR unless directly evaluated in the cited literature.

## 3 Design requirements for maternal and infant CRISPR testing

Cas12- and Cas13-based diagnostics couple sequence-specific target recognition with collateral cleavage of labeled reporters. SHERLOCK and DETECTR established the core concept, while later systems extended multiplexing, portable readout, field use, and one-pot operation (Gootenberg et al., 2017, 2018; Chen et al., 2018; Myhrvold et al., 2018; Ackerman et al., 2020; Broughton et al., 2020; de Puig et al., 2021; Fozouni et al., 2021). For maternal–infant testing, the nuclease should be treated as one module within a clinically specified system. Development should start with the clinical decision and intended specimen, then work backward through target choice, recovery, amplification, readout, controls, and workflow. In this review, a “locked test system” refers to a finalized assay configuration in which reagents, specimen-processing steps, thresholds or algorithms, readout, and operating workflow are fixed before clinical validation.

### 3.1 Target biology, sequence conservation, and inclusivity

HIV target selection requires balancing abundance, conservation, and clinical meaning (Swanson et al., 2005). Viral RNA in plasma reflects active viremia, whereas proviral DNA in cellular blood fractions can support infant diagnosis but is not equivalent to plasma viral load. Candidate regions such as *gag*, *pol*, and *LTR* differ in conservation across subtypes and recombinant forms (Lambert et al., 2003). Amplification primers and crRNA create two sequence-dependent checkpoints; a mutation in either can reduce performance (Molina Vargas et al., 2024). Multi-target designs can improve inclusivity and resilience, but they also increase reagent competition, background, and interpretation complexity. In EID, where failure at a single target site may delay treatment, *in silico* inclusivity should be followed by contrived panels and clinical specimens representing regional diversity. The CRISPR studies identified in this review were overwhelmingly HIV-1-focused, and none provided sufficient intended-use evidence for HIV-2 or combined HIV-1/HIV-2 detection. Commercial qualitative nucleic acid testing can provide combined HIV-1/HIV-2 coverage and therefore remains an important comparator where dual coverage is required (Hans et al., 2021).

HIV-focused CRISPR studies span near-patient RNA detection, digital viral-load concepts, infant diagnosis, and multiplex detection (Ackerman et al., 2020; Li et al., 2023; Nouri et al., 2023; Uno et al., 2023; Gaur et al., 2026). Related CRISPR enrichment approaches such as FLASH illustrate how Cas9-based target enrichment may improve recovery of low-abundance sequence variants, but they do not constitute HIV-specific near-patient diagnostics (Quan et al., 2019). Resistance-enrichment and mismatch-engineered systems show how guide design can improve single-nucleotide discrimination, but these capabilities are secondary to the immediate maternal–infant priorities. A genotype or resistance claim requires validation across clinically relevant allele frequencies and neighboring polymorphisms; discrimination of purified oligonucleotides alone is insufficient.

Hepatitis B virus targets in S, C, P, or X regions must account for genotypes, vaccine- or treatment-associated mutations, deletions, and overlapping reading frames. The one-pot loop-mediated isothermal amplification (LAMP)–Cas12b study detected genotypes A–F but showed materially different analytical sensitivity and lower clinical sensitivity in one genotype subgroup, demonstrating how an impressive overall estimate can mask sequence-dependent failure (Xu et al., 2024). Genotype B/C assays achieved accurate discrimination in local panels but are inherently geographically bounded (Chen et al., 2021; Zong et al., 2022). Assay development for maternal risk classification should prioritize a conserved DNA target and sufficient redundancy to reduce the risk that a single polymorphism causes threshold misclassification.

Hepatitis B virus cccDNA and pgRNA assays illustrate additional CRISPR capabilities but are not priority biomarkers for routine triple-elimination services. Their interpretation depends on pre-analytical separation of replication intermediates and on disease-state biology that is distinct from maternal HBV DNA risk classification (Allweiss et al., 2023; Zhang et al., 2022; Kongsomboonchoke et al., 2026). They are therefore considered mainly as research examples of target-specific pre-analytical complexity.

*Treponema pallidum* assays commonly target the conserved tpp47 locus (tp0574), which is referred to as tp47 in some source studies. Direct detection must consider organism burden, target copy number, and possible residual nucleic acid after treatment. Lineage and 23S rRNA resistance genotyping can be layered onto detection, as demonstrated by PCR–LwCas13a, but genotyping generally requires more target molecules than binary detection (Chen et al., 2022). An assay may therefore report the organism as detected while being unable to provide reliable resistance information; the result format should make this distinction explicit.

### 3.2 Specimen constraints and pre-analytical target recovery

Maternal and infant diagnostics are fundamentally constrained by specimen characteristics. Plasma and serum are appropriate matrices for cell-free HIV RNA and HBV DNA but require separation. Whole blood retains cell-associated proviral DNA and permits finger-stick collection, yet hemoglobin, immunoglobulins, anticoagulants, and cellular debris can inhibit amplification or nuclease reactions. Many prototype assays have been developed with purified nucleic acid, diluted matrix, or artificially spiked material; performance under those conditions should not be assumed to represent recovery from undiluted intended-use specimens.

For infant EID, the measured input volume should be treated as a design parameter rather than a procedural footnote. At low concentration, the expected number of target molecules entering a reaction follows sampling statistics; no downstream amplification can recover a molecule that was never captured. Dried blood spots offer advantages for transport and biosafety but introduce variation in spot volume, drying, degradation, punching, and elution (Sharma et al., 2014; Smit et al., 2014). A credible CRISPR EID system should report the original blood volume represented in the final reaction, extraction recovery, invalid-sample proportion, and performance after realistic storage.

Syphilis requires a specimen-specific strategy (Theel et al., 2020). Lesion swabbing should standardize lesion preparation, swab material, collection pressure, transport medium, and adequacy. Blood-based direct detection should anticipate low and intermittent target abundance (Grange et al., 2012). Placental and tissue testing require controls for tissue adequacy, inhibition, and contamination from maternal blood. Cerebrospinal fluid or amniotic fluid should not be promoted as routine specimen types merely because the molecular assay is sensitive; clinical indication and procedural risk remain decisive (Ryan et al., 2026).

The front end of the test should integrate lysis, target release, concentration where needed, inhibitor removal, and transfer into the amplification reaction. “Extraction-free” should mean validated use of the unpurified intended specimen, not omission of a commercial column after extensive heat, dilution, or magnetic processing. A rapid crude-processing method demonstrated feasibility in an HBV LAMP–Cas12a study, while centrifugal microfluidics integrated whole-blood separation and magnetic extraction in a closed disk (Ding et al., 2021; Zong et al., 2022). The latter is more representative of a true system solution, although its validation used simulated rather than patient specimens.

For decentralized use, the control strategy needs to address at least three failure domains: specimen adequacy, extraction and amplification performance, and CRISPR/readout function. A specimen-adequacy control verifies that biological material was collected. A full-process control traverses extraction, amplification, and detection, thereby revealing inhibition or reagent failure. A CRISPR/readout control verifies reporter and instrument function, while a separate negative control helps identify contamination. These functions may be implemented in separate or multiplexed channels, but each requires explicit acceptance criteria. Many publications include no-template controls during development without integrating patient-level controls into the cartridge; this is insufficient for decentralized clinical use. Supplementary Table 4 provides a structured reporting checklist for these and other complete-system variables.

### 3.3 Amplification strategies and contamination control

Pre-amplification remains the most commonly used strategy for achieving low-copy sensitivity. PCR provides high specificity and familiar quantitative behavior but requires thermal cycling and often open transfer to CRISPR (Li et al., 2021). Recombinase polymerase amplification (RPA) and recombinase-aided amplification (RAA) operate at approximately 37°C–42°C and can be rapid, yet may generate primer-dependent background, exhibit lot-dependent kinetics, and offer limited quantitative fidelity (Tan et al., 2022). LAMP can generate abundant product and support visual readout, but its multiple primers increase design complexity and non-specific amplification risk (Yang et al., 2024). T7 transcription adds another amplification layer for Cas13 detection but introduces RNA handling and an additional enzyme (Gootenberg et al., 2017).

Current platforms mainly pair CRISPR detection with PCR, RPA/RAA, or LAMP, while a smaller group uses multistage signal-amplification or rolling-circle schemes (Kaminski et al., 2021; Wang et al., 2021; Kachwala et al., 2022; Xu et al., 2024). These architectures can lower reaction-level limits of detection, but every additional enzymatic or transfer step lengthens the workflow, increases carryover risk, and makes it harder to identify where specificity is lost. RACECAR and several hairpin- or transcription-based cascades illustrate this trade-off: they produce strong signal amplification but rely on multistage procedures that are poorly aligned with decentralized maternal–infant testing (Kachwala et al., 2022; Zhao et al., 2022; Song et al., 2023).

One-pot operation is attractive because it reduces transfer steps, but achieving biochemical compatibility is difficult. Amplification enzymes and Cas nucleases can compete for target or consume intermediates, while optimal temperatures and magnesium concentrations may differ. Some successful systems use physical separation within a tube, timed release, mineral oil, gel partitioning, split guides, or sequential activation rather than truly simultaneous chemistry (Uno et al., 2023; Wang et al., 2024; Huang et al., 2025; Zhong et al., 2025; Li L. et al., 2026). Such architectures can be effective if sealed and reproducible. The term “one-pot” should not be equated with simultaneous chemistry, closed operation, or full sample-to-answer automation; studies should report whether reactions occur concurrently or sequentially and whether a manual trigger is required.

Contamination control should be engineered, not delegated to operator vigilance. Closed cartridges, unidirectional fluidics, sealed readout, preloaded reagents, uracil–DNA glycosylase-compatible amplification where feasible, and physical separation of pre- and post-amplification areas all reduce risk. Studies that transfer amplified material into Cas-reaction or lateral-flow tubes should report contamination monitoring during repeated routine use, not only clean development runs. A slightly less sensitive sealed assay may provide greater operational reliability and a lower carryover risk than a highly sensitive open cascade in a busy clinic.

### 3.4 Qualitative detection, threshold classification, and metrologically traceable quantification

Qualitative detection determines whether the target is detected above the assay’s validated detection threshold (Templer et al., 2016). This output is appropriate for HIV EID and selected syphilis direct-detection applications only when the intended specimen and clinical population are explicit (Ochodo et al., 2021). A qualitative test requires a predefined invalid category and sensitivity estimates across clinically relevant target strata (Sabi et al., 2019). For EID, performance in the lowest reference viral-load strata and after prophylaxis is more informative than an average sensitivity dominated by high-positive samples (Mallampati et al., 2017). For syphilis, the limitations and clinical interpretation of a negative result need to be reported separately for each specimen type and disease stage (Gayet-Ageron et al., 2013).

Threshold classification asks whether a concentration lies above or below a clinical action point rather than merely whether target is present (Violette et al., 2022). The output may be derived from kinetic timing, limiting dilution, digital partitioning, competitive reporters, calibrated endpoint ratios, or concentration-tuned chambers (Ramachandran and Santiago, 2021; Luo et al., 2022). Validation should focus on the interval around the decision boundary, where misclassification changes care. Studies should deliberately oversample this interval, compare results with an internationally traceable reference method, prespecify allowable bias, and incorporate lot, operator, matrix, and dilution effects. A clinically useful report may distinguish below-threshold, gray-zone, and above-threshold results, with repeat or laboratory referral rules for the gray zone. This framework is particularly relevant to maternal HBV DNA and, more narrowly, selected HIV management thresholds (Park and Pan, 2017; Abravanel et al., 2020; Ambrosioni et al., 2026).

For applications requiring a continuous result, metrologically traceable quantification requires a defined measurand, calibrators, a documented traceability chain, dynamic range, precision, linearity, dilution rules, and measurement uncertainty. Digital CRISPR can estimate concentration from the fraction of positive partitions, potentially reducing dependence on terminal signal amplitude (Nouri et al., 2023; Ngo et al., 2024; Cai et al., 2025; Song et al., 2025). Nevertheless, partition volume, occupancy assumptions, amplification efficiency, droplet loss, and classification thresholds require validation. DIMERIC showed close correlation with droplet digital PCR (ddPCR) but yielded slightly lower concentration estimates, illustrating that a digital architecture does not eliminate method bias (Cai et al., 2025).

Bulk endpoint fluorescence frequently saturates and is strongly affected by incubation time, reporter concentration, nuclease lot, and background cleavage (Avaro et al., 2025). A high R^2^ between log concentration and a fluorescence metric in buffer is not sufficient to call a system a viral-load assay (Yan et al., 2010). Real-time kinetic parameters may improve discrimination, but they also depend on amplification kinetics and temperature control. For HIV or HBV monitoring, agreement should be evaluated in log10 copies/mL or IU/mL using Bland–Altman analysis and clinically relevant error limits, in addition to correlation (Mancuso et al., 2021).

### 3.5 Readout systems, interpretation, and internal controls

Real-time fluorescence provides kinetic information and automated thresholds but requires stable optics and temperature control. Endpoint fluorescence is simpler, although borderline calls become sensitive to read time. Lateral-flow strips minimize instrumentation, but signal-off formats–in which the test line disappears when target is present–reverse the familiar visual logic and may increase errors when lines are faint (Tian et al., 2026; Xu et al., 2026; Yan et al., 2026). Wherever possible, positive and control lines should follow an intuitive signal-on design, and reader-assisted interpretation should be validated under realistic lighting and humidity.

Alternative outputs, including electrochemical, surface-enhanced Raman scattering (SERS), nanopore, glucose-meter, and photothermal systems, expand the engineering space (Nouri et al., 2020; Fang et al., 2022; Du et al., 2023; Qu et al., 2025). Most, however, remain signal-platform demonstrations rather than clinic-ready readers. If the detector requires specialized spectroscopy, precision nanopores, laser excitation, or multistep nanomaterial preparation, its near-term value is more likely to lie in mechanistic innovation or central-laboratory sensitivity than in antenatal deployment.

Smartphone imaging can reduce dedicated optics and standardize interpretation, as shown in digital HIV CRISPR and other platforms (Ngo et al., 2024; Song et al., 2025). Deep learning may improve object counting or background discrimination, but it adds requirements for device compatibility, locked algorithms, cybersecurity, data governance, and external validation. An algorithm trained on one cartridge lot, microscope configuration, or population should not be assumed transferable. The algorithm and software version form part of the regulated test system.

Regardless of readout, the result screen should distinguish positive, negative, invalid, and indeterminate outcomes. It should display the specimen type and, for quantitative tests, units and reportable range. Internal controls should not merely produce an extra line; they require acceptance criteria confirming adequate specimen input and correct function of the full biochemical chain.

### 3.6 Integrated sample-to-answer systems

A clinically mature system must integrate metered sampling, lysis, extraction or validated direct release, amplification, CRISPR recognition, signal acquisition, interpretation, quality control, and waste containment. Reports should separate hands-on from instrument time and specify storage, shelf-life, power, calibration, maintenance, and disposal requirements. Magnetofluidic and centrifugal-microfluidic prototypes have achieved greater fluidic integration than most assays, while digital, magnetic, and gel-based systems provide useful component solutions (Zong et al., 2022; Huang et al., 2023; Uno et al., 2023; Li et al., 2024; Ngo et al., 2024; Song et al., 2025). Among the systems identified in this review, none had undergone broad maternal–infant validation. A lateral-flow strip attached to an open amplification tube is therefore a portable readout, not a sample-to-answer system; likewise, a single-tube reaction starting from purified nucleic acid does not solve specimen preparation.

### 3.7 Multiplexing: shared platform versus universal triplex assay

Multiplexing can occur at three levels. Reaction-level multiplexing detects several targets in one biochemical mixture; cartridge-level multiplexing separates targets into chambers within one disposable; service-platform integration shares instruments, training, procurement, quality assurance, and data systems while retaining pathogen-specific assays. CARMEN–Cas13 demonstrated high-order multiplexing in a sophisticated laboratory format, whereas portable STI panels and duplex HIV–syphilis assays illustrate smaller, clinically oriented combinations (Ackerman et al., 2020; Low et al., 2026; Xu et al., 2026). The terminology used in this review is defined in Supplementary Table 5.

For triple-elimination services, reaction-level triplexing is constrained by incompatible clinical markers and workflows. HIV may require RNA or cell-associated DNA, HBV risk stratification requires calibrated DNA quantity, and syphilis screening remains serological while direct DNA detection is stage- and specimen-dependent. Separate cartridge chambers can accommodate different volumes, chemistries, and calibration rules, but the strongest near-term rationale lies at the service-platform level: a common reader and quality system with separately validated assays for HIV EID, HBV reflex testing, and selected syphilis add-on detection.

## 4 Pathogen-specific evidence and translational readiness

Published CRISPR diagnostic studies span analytical proof-of-concept, spiked-matrix feasibility, retrospective clinical-specimen evaluation, prospective intended-use evaluation, and implementation evidence; these stages were not treated as equivalent. Evidence stage was used to describe study maturity, whereas the author-defined five-domain framework in Section “1.1 Literature search and study selection” and Supplementary Table 1 was used to bound clinical positioning. QUADAS-2 was applied only to applicable patient-specimen diagnostic-accuracy studies. Core pathogen-specific studies are tabulated in Supplementary Table 2, with QUADAS-2 judgments in Supplementary Table 3. Accordingly, absence of prospective clinical or implementation evidence was not considered a deficiency for studies whose stated aim was analytical or engineering proof-of-concept; rather, it limited the clinical claim that could reasonably be inferred from those studies.

### 4.1 Evidence directly relevant to maternal and infant care

Direct evidence from maternal and infant populations remains limited. The infant-focused HIV-1 clade C RPA–Cas12a study is the clearest directly relevant example identified, although it used extracted plasma rather than a microvolume whole-blood or dried-blood-spot sample-to-answer workflow (Gaur et al., 2026). Among the HBV studies identified, none prospectively classified HBsAg-positive pregnant women around a prophylaxis decision boundary in traceable IU/mL. We likewise did not identify adequately powered prospective pregnancy or congenital-syphilis cohorts, or multisite operational and clinical-impact studies embedded in integrated maternal–infant services (Chen et al., 2022; Li W. et al., 2026; Low et al., 2026; Wu et al., 2026). These observations support evidence-informed development priorities rather than established indications: HIV EID has the most compelling urgent clinical rationale, HBV has the broadest clinical-specimen evidence base, and syphilis supports the narrowest, most specimen-dependent claim.

### 4.2 HIV: clinical evidence for viral-load testing and early infant diagnosis

Clustered regularly interspaced short palindromic repeats approaches to HIV include isothermal and one-pot RNA assays, digital partitioning, multiplex surveillance, and infant-focused testing (Li et al., 2023; Nouri et al., 2023; Uno et al., 2023; Gaur et al., 2026). These studies address different analytes and clinical questions, so their performance should not be combined into a single readiness claim. Most systems are HIV-1-specific, and current evidence remains insufficient for HIV-2 or combined HIV-1/HIV-2 molecular detection.

The CARMEN–Cas13 platform illustrates the high-throughput end of the multiplexing spectrum. Color-coded sample and detection droplets enabled more than 30,000 parallel reactions across broad viral panels. In the clinical evaluation, 11,236 of 11,268 interpretable sample–assay result pairs agreed with next-generation sequencing; these pairwise results were generated from a substantially smaller number of clinical specimens and should not be interpreted as 11,268 independent patient tests. HIV drug-resistance calls showed 86%–90% agreement and improved to 93% after targets with more than three mismatches were excluded (Ackerman et al., 2020). The findings demonstrate the potential of computationally designed multi-guide surveillance and the effect of sequence mismatch. Because the workflow requires pre-amplification, droplet pairing, electrofusion, microscopy, and computational analysis, its likely role is in reference laboratories or public-health surveillance rather than near-patient maternal–infant testing.

CARRD amplifies the Cas13a signal rather than the target nucleic acid. An anti-tag hairpin feedback circuit lowered the analytical LOD for HIV and hepatitis C virus (HCV) RNA to 10 aM and increased signal at 100 fM HIV RNA by approximately 23-fold relative to conventional Cas13a detection (Moon et al., 2025). In 30 HIV plasma specimens, the assay showed group discrimination with an area under the receiver operating characteristic curve of 0.9709. This result does not establish population-level clinical sensitivity or specificity, and the workflow still depended on RNA extraction and fluorescence readout. Translation will require control of hairpin leakage and evidence that reagent manufacturing and environmental variation do not destabilize the signal. The anti-tag-mediated cascade architecture is illustrated in Figure 3A (Moon et al., 2025).

**FIGURE 3:**
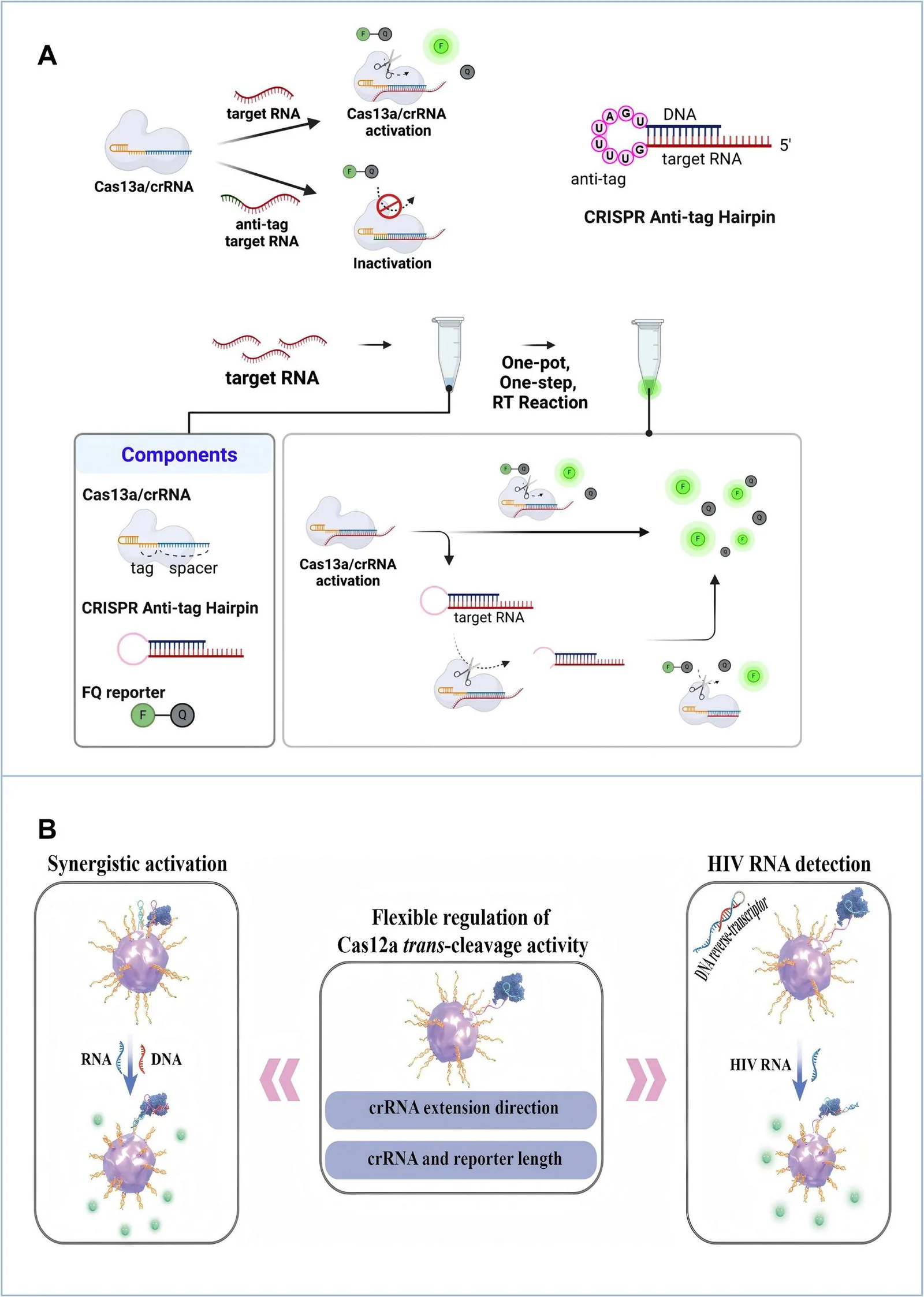
Representative HIV-directed CRISPR architectures for signal amplification and activity regulation. **(A)** CARRD (CRISPR anti-tag-mediated room-temperature RNA detection) exploits anti-tag-mediated inhibition and a CRISPR anti-tag hairpin to generate a one-pot Cas13a cascade, enabling amplification-free RNA detection at room temperature (Moon et al., 2025). Reproduced from Moon et al. (2025) Copyright © The Author(s) 2025; licensed under the Creative Commons Attribution-NonCommercial-NoDerivatives 4.0 International License (CC BY-NC-ND 4.0). **(B)** CAS-FLIER (CRISPR/Cas12a on fluid membranes for activity regulation) spatially confines Cas12a, CRISPR RNA (crRNA), and reporter molecules on a fluid membrane; crRNA extension direction and length, together with reporter length, regulate trans-cleavage, and a DNA reverse-transcriptor converts HIV RNA recognition into Cas12a activation (Yang et al., 2026). Adapted from Yang et al. (2026) Copyright © The Author(s) 2026; distributed under the Creative Commons Attribution 4.0 International License (CC BY 4.0). F/Q, fluorophore/quencher reporter; HIV, human immunodeficiency virus; RNA, ribonucleic acid.

Among the HIV studies identified in this review, amplification-based assays include some of the larger patient-specimen evaluations outside the infant-focused EID study. Li et al. (2023) evaluated an RT-RAA–Cas13a assay in 158 clinical plasma samples and reported 91.81% sensitivity and 100% specificity, with the lowest detected viral load reported as 112 copies/mL. CRISPR gel was evaluated in a much smaller set of clinical plasma samples, while other RAA/RPA–CRISPR and magnetofluidic systems illustrate attempts to shorten or contain the workflow (Zhao et al., 2021; Uno et al., 2023; Li et al., 2024). These results support clinical detection feasibility, but they do not establish EID performance because they begin with plasma or extracted nucleic acid, are not designed around prophylaxis-exposed infants, and do not validate microvolume whole-blood or dried-blood-spot recovery. The relevant comparison is therefore not amplification versus no amplification, but whether the complete system recovers and correctly classifies low-copy target from the intended specimen without unacceptable bias or carryover.

Mechanism-focused HIV studies–including signal cascades, magnetofluidic components, nanoparticle counting, and activity-regulation strategies–provide useful engineering concepts but should not be treated as independent evidence of an HIV clinical claim without patient-specimen recovery, subtype coverage, and complete-workflow validation (Huang et al., 2023; Zhang et al., 2024; Li et al., 2025; Yan et al., 2026; Yang et al., 2026). A representative spatial-confinement activity-regulation architecture is shown in Figure 3B (Yang et al., 2026).

Maternal viral-load measurement remains the least mature of the priority HIV applications. STAMP used membrane-based picoliter partitioning to convert direct Cas13a activity into countable events over a synthetic-RNA range of 1 fM to 10 pM (Nouri et al., 2023). In spiked plasma, measured concentration closely tracked the expected input, but only about 50 nL of the extracted RNA entered a membrane. Among 20 patient plasma specimens, high-positive and negative samples were generally concordant with RT-PCR, whereas low-positive samples were vulnerable to subsampling noise; the reported sensitivity was 60%, specificity was 100%, and the estimated end-to-end specimen-level detection limit was approximately 2,000 copies/mL. STAMP therefore demonstrates amplification-free digital quantification, while also showing that effective analytical volume–not Cas13a chemistry alone–sets the low-load ceiling.

Other digital approaches shift rather than eliminate complexity. A smartphone RT-RPA–Cas12a prototype counted positive microwells within 15 min and detected 75 RNA copies per reaction, but it was evaluated only with synthetic targets and the early readout captured fewer positive wells than the endpoint (Ngo et al., 2024). dCRISTOR converted dCas9 target binding into changes in magnetic micromotor velocity and classified nine patient plasma specimens correctly relative to a 1,000 copies/mL threshold (Song et al., 2025). That result is encouraging but too small to establish diagnostic accuracy, and the output is particle-level binary classification rather than classical partition-based absolute quantification. Together, these studies show that digital readout can support threshold decisions, but calibration, effective input volume, algorithm locking, and agreement analysis remain indispensable.

Subtype inclusivity is another readiness criterion. Several systems target conserved HIV-1 regions and test limited genotype panels, whereas the infant RPA–Cas12a assay focused specifically on clade C and the quantum-dot lateral-flow system was designed around CRF07_BC (Gaur et al., 2026; Yan et al., 2026). These are appropriate regional strategies, but maternal programmes need either region-specific validation or multi-target coverage. A platform that performs well in a locally predominant subtype may be clinically useful; it should carry a regionally bounded intended-use claim and a surveillance plan for target-site variation.

Current evidence does not establish that a CRISPR assay can replace a validated maternal HIV viral load platform. A near-term role would be investigational urgent triage when central results are clinically delayed, followed by standard quantification where required. Evaluation should report bias and agreement in log10 copies/mL and determine whether earlier results change perinatal management.

Early infant diagnosis has a clearer clinical rationale and a narrower tolerance for error than maternal viral-load triage. Gaur et al. (2026) evaluated a single-tube RPA–Cas12a assay targeting the HIV-1 clade C pol region in 41 seropositive plasma specimens, including 28 from children younger than 2 years. Of the 28 infant specimens, 27 were positive and one was inconclusive. Retaining the inconclusive result in the denominator gives a positive-result proportion of 96.4% (27/28; exact 95% CI, 81.7%–99.9%); the overall evaluation reported 96% sensitivity and 92.65% specificity. The study is directly relevant to EID, but it does not yet establish a deployable infant test. The lowest detected clinical sample was estimated at approximately 3 × 10^3^ copies/mL without a formal probabilistic LOD. The workflow required plasma RNA extraction and reverse transcription, was not evaluated with dried blood spots or microvolume whole blood, and provides regionally bounded evidence for clade C. Future studies should prespecify repeat rules for the assay’s inconclusive interval and report initial, repeated, and final specimen-level classifications with transparent denominators.

Several platforms provide components that could be integrated into a clinically oriented EID system. Magnetofluidic processing can reduce manual extraction, CRISPR gel and sequential systems can contain amplification and recognition, lateral-flow or quantum-dot counting can simplify readout, and digital Cas13a can provide count-based interpretation (Li et al., 2023, 2024; Nouri et al., 2023; Uno et al., 2023; Huang et al., 2025; Yan et al., 2026). The central engineering challenge is not the absence of promising modules but their integration without losing low-copy recovery, increasing invalid rates, or adding manufacturing and operator fragility.

Proviral DNA and RNA present different opportunities. Cell-associated proviral DNA can be detected from whole blood and is compatible with infant diagnosis, whereas RNA reflects active viremia but may require plasma separation or robust whole-blood handling. Live-cell proviral imaging is a research tool rather than an EID pathway (Ma et al., 2017). Assays detecting plasma RNA, cell-associated proviral DNA, and total HIV nucleic acid should not be pooled under a single EID performance claim; each analyte has different pre-analytical requirements and may respond differently to maternal or infant antiretroviral exposure. The reference method and clinical interpretation should be aligned with the stated analyte.

Future EID validation should use the intended microvolume whole-blood and/or dried-blood-spot workflow, report low-positive performance together with initial invalid, repeat, discordant, and final classifications, and incorporate analyte-appropriate specimen-adequacy and full-process controls. Detailed development specifications are summarized in Table 2.

**TABLE 2:** Evidence-informed development and validation specifications for priority clinical applications, with source basis indicated in each cell.

Specification domain	HIV EID	Maternal HBV reflex classification	Syphilis adjunct direct detection
Clinical decision/comparator	[G] Diagnose HIV infection in an exposed infant to enable rapid ART; comparator: validated qualitative NAT.	[G/M] Classify prophylaxis eligibility after HBsAg positivity; comparator: IU/mL-traceable laboratory HBV DNA or a locally accepted surrogate pathway.	[G/A] Add pathogen evidence in defined early/discordant presentations; comparator: treponemal/non-treponemal serology plus clinical assessment.
Intended-use population	[G/A] HIV-exposed infants; prespecify representation of low-positive and prophylaxis-exposed infants.	[G/A] Consecutive HBsAg-positive pregnant women, enriched around the locally applicable prophylaxis boundary.	[G/A] Defined presentation, initially compatible early lesions or discordant serology; pregnancy/neonatal claims require dedicated cohorts.
Specimen and input	[G/M] Metered microvolume whole blood; separate validation for dried blood spots or plasma.	[M] Whole blood, plasma, or serum; document original volume, recovery, and dilution.	[M/A] Lesion/mucosal swab for an initial claim; blood, placental/cord/other tissue, or neonatal samples require separate claims.
Primary output	[G/M] Positive / negative / invalid / indeterminate qualitative result; prespecified repeat rule for indeterminate results.	[G/M/A] Below threshold / gray zone / above threshold / out of range in IU/mL; gray zone triggers repeat or traceable laboratory testing.	[M/A] Positive / negative / invalid / indeterminate result; a negative result must state that it does not exclude syphilis or replace indicated serological/clinical assessment.
Comparative performance	[M/A] Prespecified comparison with standard NAT; report 2 × 2 counts and confidence intervals plus invalid, repeat, and discordant-result handling.	[M/A] Estimate boundary-specific classification error and allowable bias; report agreement rather than correlation alone.	[M/A] Report specimen- and stage-specific accuracy with exact confidence intervals and incremental value beyond serology.
Controls	[M/A] Specimen-adequacy, non-competitive full-process, and readout controls.	[M/A] Full-process, extraction/inhibition, high-load/dilution, and negative controls.	[M/A] Matrix-specific specimen-adequacy, full-process, readout, and contamination controls.
Inclusivity and interference	[M/A] Regional HIV-1 subtype/recombinant inclusivity; antiretroviral exposure; matrix interference.	[M/A] Genotype/target-site inclusivity; HBeAg status; matrix interference; high-load effects.	[M/A] Disease/treatment stage; related treponemes/organisms; blood contamination; matrix interference.
Operational endpoints	[M/A] Total/hands-on time; initial invalid/repeat rates; result receipt; time to ART.	[M/A] Same-visit result; timely prophylaxis; missed/unnecessary prophylaxis; no delay to infant vaccination.	[M/A] Time to treatment; resolution of discordance; repeat testing; false reassurance/unnecessary therapy.
Product robustness	[M/A] Shelf-life/stability across a prespecified storage/transport range; power/connectivity; reader downtime; lot/operator reproducibility.	[M/A] Calibration stability; lot/operator effects; high-load/out-of-range handling; storage/transport; connectivity; referral rules.	[M/A] Storage/transport stability; closed operation; reader interpretation; specimen-collection training; report-language safeguards.

Combined tags indicate cells with more than one source basis; the tags identify source basis, not an evidence grade or hierarchy. These development and validation specifications are intended for translational research and are not regulatory, WHO-endorsed, or consensus target product profiles. Numerical acceptance criteria should be prespecified for the intended population, comparator pathway, and consequence of error. ART, antiretroviral therapy; EID, early infant diagnosis; HBeAg, hepatitis B e antigen; HBsAg, hepatitis B surface antigen; HBV, hepatitis B virus; HIV, human immunodeficiency virus; IU/mL, international units per milliliter; NAT, nucleic acid testing. Source basis: [G], guideline or established clinical pathway; [M], published methodological or diagnostic-performance principle; [A], author-proposed translational specification.

Current evidence does not support replacement of standard nucleic acid testing (NAT) for EID. Qualitative infant diagnosis is nevertheless more closely aligned with current CRISPR capabilities than full maternal viral-load quantification, making EID a compelling near-term development opportunity provided that intended-specimen recovery, HIV-1 diversity, prophylaxis exposure, integrated controls, multisite reproducibility, and linkage to timely treatment are prospectively validated.

### 4.3 HBV: from DNA detection to clinically actionable classification

Clinical HBV evidence is broader than that for HIV EID or syphilis direct detection, spanning large clinical detection studies, low-level-viremia cohorts, one-pot clinical assays, and digital measurement (Wang et al., 2021; Tian et al., 2023; Xu et al., 2024; Cai et al., 2025). Wang et al. (2021) combined PCR, T7 transcription, and Cas13a for HBV DNA detection and YMDD-motif genotyping. In a blinded set of 280 serum specimens, positive percent agreement with qPCR was 100% (90/90; exact 95% CI, 96.0%–100%) and negative percent agreement was 94.7% (180/190; exact 95% CI, 90.5%–97.4%); low-level ddPCR signals supported some discordant results but did not fully adjudicate every specimen. The open multistage workflow and unresolved discordance limit translation to maternal reflex testing.

Several clinical studies published in 2026 further reinforce the distinction between detection feasibility and maternal decision validity. Sata et al. (2026) applied RPA/nested-RPA–Cas13a to 45 human clinical samples in the HBV cohort of an Indian study. A dual Cas12a/Cas13a PCR-CRISPR platform evaluated 46 serum samples from untreated and treated patients with chronic HBV infection; the authors reported 97.06% sensitivity and 100% specificity for HBV DNA relative to an ultrasensitive qPCR comparator (Li Y. et al., 2026). A light-activated one-pot enzymatic recombinase amplification (ERA)–Cas12a platform evaluated 79 clinical serum samples; the authors reported 100% sensitivity by fluorescence and 96.3% by lateral flow, with strong agreement with qPCR (Zhou et al., 2026). A dual HBV/hepatitis D virus (HDV) assay evaluated 70 plasma specimens from participants with HBV/HDV coinfection, with positive concordance of 85.7% (60/70) by fluorescence and 82.9% (58/70) by lateral flow (Tian et al., 2026). These studies strengthen evidence for clinical detection and workflow integration, but none constitutes prospective, boundary-focused validation in HBsAg-positive pregnant women with IU/mL-traceable classification.

Tian et al. (2023) studied a clinically defined low-level-viremia population rather than a high-contrast case–control panel. The RAA–T7–Cas13a lateral-flow assay was evaluated in 180 participants with HBV infection, including 100 with low-level viremia, and in 111 longitudinal plasma specimens from 23 treated patients. Positive percent agreement with qPCR was approximately 87% in the low-level-viremia group and reached 100% above 100 IU/mL. In longitudinal specimens, positive and negative percent agreement were 100% and 92.15%, respectively. Four CRISPR-positive/qPCR-negative results were not fully adjudicated with an orthogonal high-sensitivity method. The findings illustrate how original specimen volume and stochastic sampling may influence performance below 100 IU/mL and reinforce the distinction between qualitative detection and standardized viral load measurement.

Apparently perfect accuracy in small or spectrum-restricted panels warrants caution. Performance can be inflated by selecting unequivocal positives and healthy controls, optimizing thresholds on the evaluation dataset, or omitting invalid results. Across the HBV literature, aggregate detection accuracy should therefore be interpreted alongside specimen recovery, original input volume, genotype distribution, comparator limitations, and the number and disposition of invalid or discordant results.

Moving from qualitative detection to digital measurement, DIMERIC is among the clearest examples of digital HBV measurement (Cai et al., 2025). The chip separated a 15-min RPA step from a 5-min Cas12a detection step and then electrofused paired droplets, avoiding direct biochemical competition between amplification and nuclease activity. Dilution-series measurements were highly linear (R^2^ = 0.99893). In 24 clinical serum specimens, qualitative results agreed across DIMERIC, qPCR, and ddPCR; the coefficients of determination were R^2^ = 0.92033 versus qPCR and R^2^ = 0.97337 versus ddPCR. DIMERIC nevertheless produced slightly lower concentration estimates. Association does not establish interchangeability, and the study did not report Bland–Altman limits of agreement or replicated performance near a maternal decision boundary. The reported 20-min on-chip reaction also excluded extraction, emulsification, loading, imaging, and analysis. Polydimethylsiloxane (PDMS) microfluidics, controlled droplet pairing, electrofusion, and microscopy remain barriers to decentralized maternal testing. The study established the technical feasibility of digital count-based HBV measurement, not interchangeability with a traceable clinical viral load assay.

Hepatitis B virus HOLMESv2 combined LAMP and AapCas12b at 60 °C in a single-tube reaction (Xu et al., 2024). Across 236 clinical specimens, it detected 198/200 qPCR-positive samples (99.0%; exact 95% CI, 96.4%–99.9%) and 36/36 qPCR-negative samples (100%; exact 95% CI, 90.3%–100%). Fluorescence threshold time correlated with concentration across approximately six orders of magnitude. Subgroup analysis identified a potential inclusivity limitation: detection was 16/16 in genotype C samples and 7/9 in genotype B samples, and both false negatives contained three guide-target-region mutations. Because the genotype B subgroup was small, this is an inclusivity signal rather than a general performance estimate. Conversion to approximately 25 copies/mL assumed ideal extraction recovery, and quantification was not traceable to international units. The study demonstrates single-tube clinical feasibility while showing that aggregate accuracy can mask target-mismatch-associated failure and kinetic bias.

Alternative HBV readouts–including electrochemical, SERS, nanopore, glucose-meter, and nanoparticle-based systems–have produced concentration-responsive signals under controlled conditions (Fang et al., 2022; Wang et al., 2022; Du et al., 2023; Zhao et al., 2024). Most have not undergone patient-level agreement studies, internationally traceable calibration, or validation of a clinical reportable range. They should therefore be described as quantitative signal platforms rather than HBV viral-load assays. A deployable system must also handle both extremes of the clinical range: high viremia may saturate bulk signals, whereas low viremia is dominated by stochastic sampling and extraction loss. Automatic dilution, threshold-tuned chambers, or digital partitioning may address these problems, but results outside the validated range must be reported without false precision. Representative HBV architectures span cccDNA-specific pre-analytical processing and Cas13a readout (Figure 4A; Zhang et al., 2022), integrated centrifugal whole-blood processing with RPA-T7-Cas13a detection (Figure 4B; Zong et al., 2022), multistage RPA-CRISPR-HCR signal amplification (Figure 4C; Kachwala et al., 2022), and LAMP-Cas12a detection with fluorescence or lateral-flow readout (Figure 4D; Ding et al., 2021).

**FIGURE 4:**
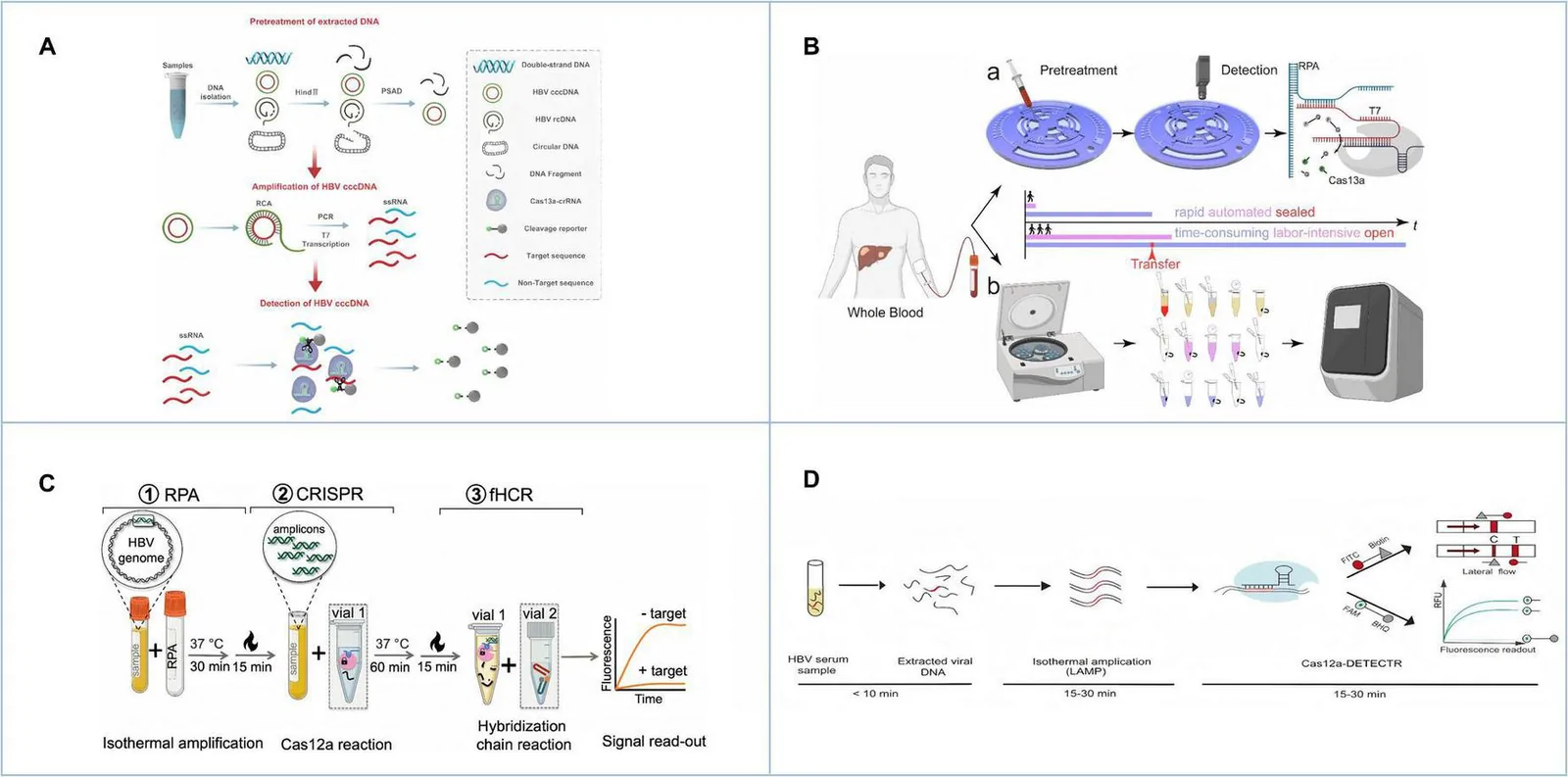
Representative HBV CRISPR architectures spanning target specificity, sample integration, signal amplification, and rapid readout. **(A)** An HBV covalently closed circular DNA (cccDNA) assay combines HindIII/plasmid-safe ATP-dependent DNase (PSAD) pretreatment, rolling-circle amplification (RCA)/PCR, T7 transcription, and Cas13a fluorescence, illustrating the dependence of cccDNA specificity on pre-analytical processing (Zhang et al., 2022). Adapted from Zhang et al. (2022) Copyright © The Author(s) 2022; distributed under CC BY 4.0. **(B)** A centrifugal microfluidic platform integrates whole-blood separation, magnetic nucleic-acid extraction, and recombinase polymerase amplification (RPA)-T7-Cas13a genotyping in a sealed disk (Zong et al., 2022). Adapted with permission from Zong et al. (2022) Copyright © 2022 American Chemical Society. **(C)** Recombinase amplified CRISPR enhanced chain reaction (RACECAR) couples RPA, Cas12a, and fluorescence hybridization chain reaction (fHCR) in sequential amplification stages for HBV detection (Kachwala et al., 2022). Adapted with permission from Kachwala et al. (2022) Copyright © The Royal Society of Chemistry 2022. **(D)** A loop-mediated isothermal amplification (LAMP)-Cas12a DNA Endonuclease-Targeted CRISPR Trans Reporter (DETECTR) workflow combines rapid serum processing with isothermal amplification and fluorescence or lateral-flow readout (Ding et al., 2021). Adapted from Ding et al. (2021) Copyright © 2021 by the authors; distributed under CC BY 4.0. HBV, hepatitis B virus; PCR, polymerase chain reaction.

For maternal risk classification, analytical sensitivity below the intervention threshold is necessary but not sufficient. The decisive property is correct classification near the locally applicable action boundary across lots, operators, genotypes, and matrices. Broad-range correlation or detection far below the threshold does not demonstrate low boundary misclassification.

A clinically oriented classifier could report below-threshold, gray-zone, and above-threshold results, with predefined out-of-range and referral rules. Such a configuration remains a development concept; prospective validation would require an IU/mL-traceable reference and prespecified boundary-focused performance criteria (Table 2).

Hepatitis B virus pgRNA and cccDNA assays demonstrate additional research capabilities (Zhang et al., 2022; Allweiss et al., 2023; Zhu et al., 2025; Chen et al., 2026; Kongsomboonchoke et al., 2026), but their biological interpretation and pre-analytical complexity make them unsuitable as near-term triple-elimination priorities.

Hepatitis B surface antigen protein detection and infant molecular testing also require bounded claims. Nucleic acid testing cannot assess vaccine-induced anti-HBs, and an early positive DNA result cannot define persistent infection; exposed infants still require appropriate prophylaxis and longitudinal serological and, where indicated, molecular follow-up.

Hepatitis B virus relatively abundant, stable DNA and broader clinical-specimen evidence have not yet yielded validated maternal threshold classification. Progress depends chiefly on traceability, genotype inclusivity, high-load control, gray-zone handling, and evidence that same-visit reflex testing improves timely prophylaxis without delaying vaccination.

### 4.4 Syphilis: specimen-dependent direct detection within a serology-led pathway

The principal CRISPR evidence for syphilis comes from a sensitive PCR–T7–LwCas13a clinical benchmark, portable or one-pot clinical evaluations, an RPA–Cas12a blood/serum study, and a duplex HIV–syphilis format (Chen et al., 2022; Li W. et al., 2026; Low et al., 2026; Wu et al., 2026; Xu et al., 2026). Together, these studies show a consistent trade-off: laboratory amplification and fluorescence can maximize analytical sensitivity, whereas isothermal, single-tube, or strip-based formats reduce instrumentation but remain vulnerable to low organism burden and specimen-processing loss. Representative architectures include RPA–Cas12a detection (Figure 5A; Li W. et al., 2026), amplification-free Cas13a–DNAzyme signal amplification (Figure 5B; Yin et al., 2026), and PCR–T7–LwCas13a detection and genotyping (Figure 5C; Chen et al., 2022).

**FIGURE 5:**
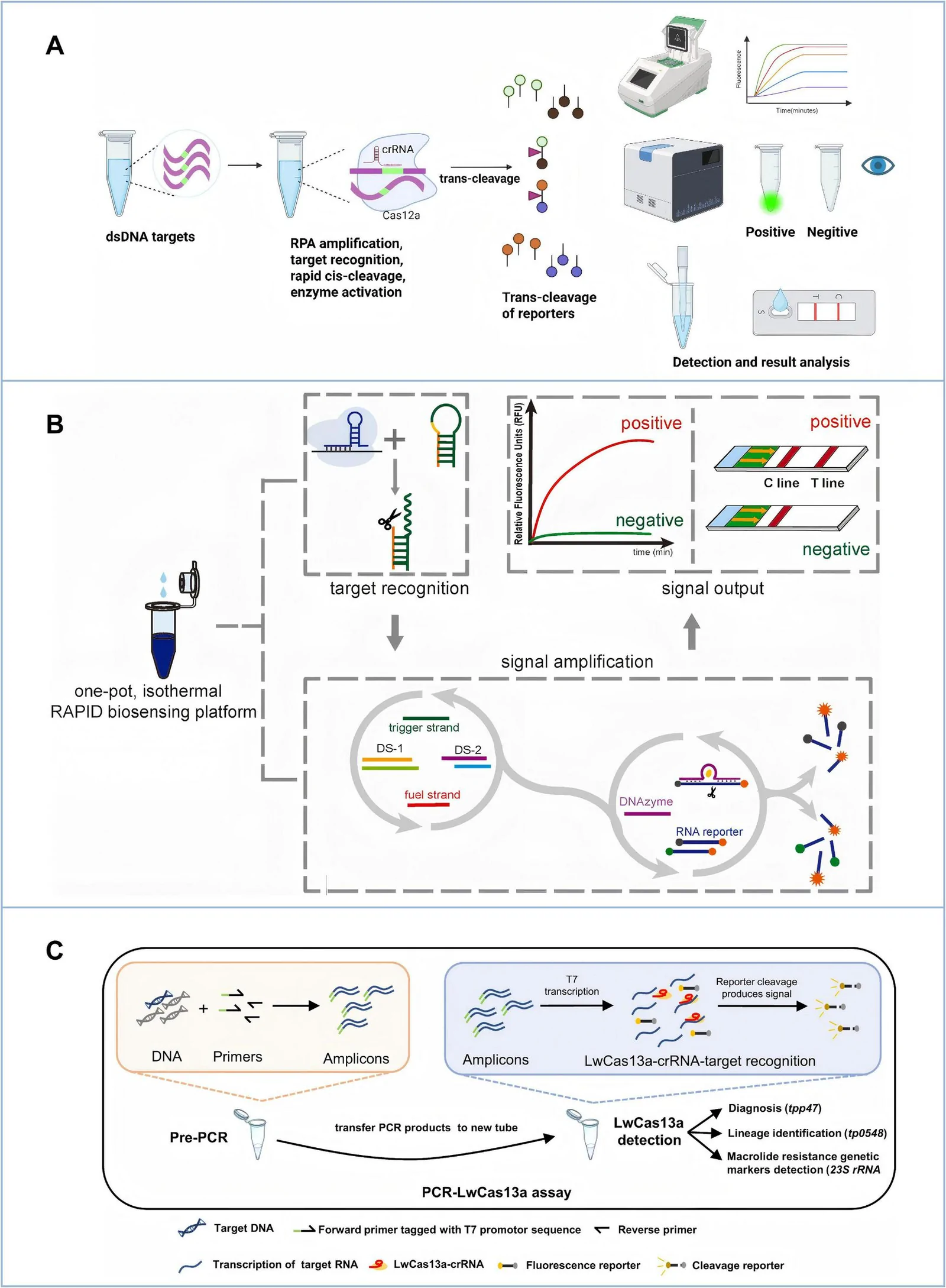
Representative CRISPR architectures for direct *T. pallidum* detection and molecular characterization. **(A)** A recombinase polymerase amplification (RPA)-Cas12a workflow amplifies the tpp47 locus (tp0574; reported as tp47 in the source study) and uses Cas12a collateral cleavage for fluorescence, direct visual, or lateral-flow readout (Li W. et al., 2026). Reproduced from Li W. et al. (2026) Copyright © The Author(s) 2026; licensed under CC BY-NC-ND 4.0. **(B)** RAPID (CRISPR-Cas13a with a cascade amplification circuit-driven DNAzyme) couples Cas13a target recognition to a toehold-mediated strand-displacement DNA circuit and DNAzyme cascade, providing one-pot fluorescence and lateral-flow outputs; *T. pallidum* polA messenger RNA (mRNA) was evaluated as a proof-of-concept target (Yin et al., 2026). Reproduced from Yin et al. (2026) Copyright © 2026 The Author(s); published by Elsevier B.V.; licensed under CC BY-NC-ND 4.0. **(C)** PCR-LwCas13a (Leptotrichia wadei Cas13a) uses T7-tagged PCR products, transcription, and Cas13a reporter cleavage for *T. pallidum* detection, lineage identification, and 23S ribosomal RNA (rRNA) macrolide-resistance genotyping (Chen et al., 2022). Adapted from Chen et al. (2022) Copyright © The Author(s) 2022; distributed under CC BY 4.0. PCR, polymerase chain reaction.

Chen et al. (2022) provide the most detailed clinical benchmark for CRISPR-based *T. pallidum* detection. Their PCR–LwCas13a assay was evaluated in 216 specimens from 135 people with primary or secondary syphilis and 81 controls. In whole blood, sensitivity was 71.4% in primary and 87.7% in secondary syphilis, compared with 28.6% and 45.6%, respectively, for tpp47 qPCR. Skin biopsies and genital lesion swabs showed substantially higher detection rates than whole blood with both methods. Across the mixed specimen set, CRISPR sensitivity was 93.3% and specificity was 100%; among 41 specimens assessed by rabbit infectivity testing, detection rates were 87.8% for PCR–LwCas13a, 85.4% for infectivity testing, and 61.0% for qPCR. The assay also distinguished major lineages and detected A2058G/A2059G macrolide-resistance mutations. These results demonstrate broad clinical capability, but the aggregate performance estimate should not be extrapolated to pregnancy, latent infection, or congenital syphilis. The workflow still required PCR, open transfer, T7 transcription, and approximately 75 min, placing it closer to an enhanced laboratory test than to a decentralized maternal assay.

Li W. et al. (2026) evaluated 30 clinically characterized specimens from people with suspected syphilis; the report describes peripheral venous blood collection but later refers to serum in the clinical validation. Operators were blinded to clinical status during index testing, and the authors reported 96.6% overall diagnostic accuracy, reflecting high concordance with the study’s serology-based clinical reference diagnosis (Li W. et al., 2026). This study is useful evidence for blood-derived direct detection, but it remains small, required automated nucleic-acid extraction, and does not validate the higher-yield lesion/mucosal-swab claim or a pregnancy-specific population.

Low et al. (2026) evaluated a portable dual-target multiplex format using 900 masked, extracted-DNA specimens from several anatomical sites; 400 specimens entered the *T. pallidum*–herpes simplex virus (HSV) arm. *T. pallidum* sensitivity was 82.5%, and 17/18 initially false-negative specimens became positive on repeat testing. This pattern is consistent with stochastic low-copy sampling and run-to-run variability, although the dominant failure mechanism was not established. The approximately 55-min assay used portable fluorescence and dual targets, but it began with laboratory-extracted DNA and was evaluated retrospectively at a reference laboratory. Repeat testing may recover some low-load positives, but it increases cost and complicates same-visit interpretation.

A smaller duplex study by Xu et al. (2026) integrated reverse transcription, multiplex RPA, transcription, Cas12a/Cas13a detection, and a dual-line lateral-flow readout in a one-pot format. In 46 clinical samples, the authors reported 100% sensitivity and 97.5% specificity for HIV and 90% sensitivity and 100% specificity for *T. pallidum* relative to qPCR, with an analytical detection limit of 100 copies/μL and a 45-min workflow. This study demonstrates clinically oriented duplex chemistry and reagent lyophilization, but the sample set was small, the two targets serve different clinical pathways, and the line-elimination readout and extraction-free lysis require independent usability and intended-specimen validation. It supports a cartridge-engineering concept rather than a universal antenatal or maternal–infant screening claim.

Wu et al. (2026) integrated RPA, T7 transcription, and Cas13a in a single 37 °C tube and evaluated 186 specimens from four centers, including whole blood, lesion exudate, and cerebrospinal fluid. The fluorescence assay reported an analytical LOD of 10 copies/μL and an estimated total turnaround time of about 50 min including extraction. Specificity was reported as 100% across specimen types, and lesion exudate showed the highest sensitivity. The lateral-flow version required substantially more target, with a practical LOD of approximately 200 copies/μL. These estimates should not be converted into specimen-level sensitivity without the original specimen volume, extraction recovery, and reaction input. Single-tube chemistry reduces transfer risk, but it does not overcome low organism abundance in blood or cerebrospinal fluid, nor does it remove extraction, storage, or reader requirements.

Specimen choice is a major determinant of direct-detection yield. Blood is convenient in antenatal services but may contain too few organisms for a reliable exclusion test. Lesion swabs usually provide a higher-yield matrix, although they are available only during selected disease stages and depend on correct lesion recognition and sampling. Placental, cord, tissue, cerebrospinal-fluid, and neonatal-lesion claims require independent validation because contamination, invasiveness, tissue adequacy, and reference-standard uncertainty differ from those of blood or lesion swabs. Clinical studies should therefore report each matrix separately, using a specimen-specific threshold, reference standard, and intended interpretation. When participant numbers are small, raw 2 × 2 data with exact confidence intervals are more informative than a pooled headline sensitivity.

Beyond specimen matrix, disease stage substantially influences direct-detection yield. Primary and secondary syphilis are the most suitable candidates because lesions or higher organism burden may be present. Early latent, late latent, or treated infection may yield negative direct tests despite clinically important disease history or ongoing management needs. Conversely, detection of *T. pallidum* nucleic acid after treatment does not necessarily demonstrate organism viability, treatment failure, or ongoing transmissibility because the duration of residual nucleic acid detectability is not well defined. Congenital syphilis is especially complex: infection may involve the placenta and multiple fetal tissues, but accessible neonatal blood may contain a low organism burden and maternal antibodies confound serology. Current CRISPR evidence is insufficient to define sensitivity or negative predictive value in a large prospective congenital syphilis cohort.

Resistance and lineage detection add epidemiological value but should not distract from treatment during pregnancy. Penicillin remains the critical therapy for preventing congenital syphilis, whereas the macrolide-resistance mutations detected by the PCR–LwCas13a study are more relevant to surveillance and non-pregnancy treatment considerations (Chen et al., 2022). A test cartridge for maternal care should prioritize robust organism detection and specimen adequacy over optional genotyping unless the latter can be added without sacrificing sensitivity or increasing failure.

Within maternal and neonatal care, the most defensible near-term claim is specimen-specific add-on detection, particularly from a compatible lesion when serology is non-reactive or discordant, provided that indicated treatment is not delayed. Placental, cord, tissue, and neonatal-specimen claims remain investigational and require separate validation.

Negative results require explicit non-exclusion language because non-detection can reflect lesion age, sampling quality, prior treatment, or low and intermittent organism burden. Current evidence therefore does not support universal antenatal molecular screening or stand-alone exclusion of congenital infection.

### 4.5 Comparative synthesis of pathogen-specific evidence and translational gaps

Clinical priority and evidentiary maturity diverge across applications. HIV EID has the strongest urgent clinical rationale but remains limited by microvolume recovery, low target abundance, prophylaxis exposure, and viral diversity; maternal HBV has the broadest clinical-specimen evidence but lacks traceable, boundary-focused pregnancy validation; and syphilis direct detection supports the narrowest, specimen- and stage-dependent claim. Across all three, incomplete specimen integration and limited intended-use validation are more important constraints than nuclease chemistry.

Table 3 therefore compares evidence status across the five appraisal domains, the principal translational gaps, and the narrowest defensible clinical position rather than assigning a readiness score.

**TABLE 3:** Comparative evidence status and translational positioning of selected CRISPR applications and the universal-triplex concept.

Selected application	Direct intended-use evidence	Specimen integration	Measurement/ interpretation	Control/containment	Operational/ clinical-utility evidence	Evidence-informed clinical position
HIV EID	Limited direct evidence: infant-focused clade C plasma study, with specimen/workflow mismatch (Gaur et al., 2026).	Major gap: no validated microvolume whole-blood or dried-blood-spot sample-to-answer workflow.	Qualitative output fits EID; low-positive and prophylaxis-stratified performance remain inadequately characterized.	Full-process controls, invalid handling, containment, and multisite robustness remain unproven at product level.	No CRISPR-specific intended-user or multisite evidence linking testing to faster ART initiation.	Priority for intended-use validation; insufficient evidence to replace validated NAT.
Maternal HIV viral load	No direct intended-use evidence identified. Supporting evidence: small patient studies and digital proof-of-concept platforms (Nouri et al., 2023; Ngo et al., 2024; Song et al., 2025).	Mostly extracted RNA; effective analyzed volume is often small.	Association or threshold concepts are shown; traceable agreement across the clinical range is not established.	Calibration, software, effective-volume, and high-range error controls remain unresolved.	No evidence identified that CRISPR testing improves peripartum decision timing or patient-level outcomes.	Investigational triage only; insufficient evidence to replace calibrated quantitative viral-load testing.
Maternal HBV reflex classification	No direct evidence for prospective pregnancy threshold classification. Supporting evidence: multiple clinical serum/plasma studies (Wang et al., 2021; Tian et al., 2023; Xu et al., 2024; Cai et al., 2025).	Serum/plasma workflows are common; whole-blood integration is emerging.	Detection is well supported; IU/mL traceability and boundary misclassification remain unresolved.	Single-tube/integrated formats exist; high-load, genotype, gray-zone, and lot/operator controls need validation.	No prospective evidence identified that same-visit CRISPR classification improves timely prophylaxis or reduces referral loss.	Broadest clinical-specimen evidence base, but a clinical claim requires traceable, boundary-focused pregnancy validation.
Syphilis adjunct direct detection	No direct evidence for pregnancy or congenital intended use. Supporting evidence: lesion, blood, and other-matrix clinical studies, mainly in early disease (Chen et al., 2022; Low et al., 2026; Wu et al., 2026).	Lesion specimens are higher yield; blood/tissue performance is inconsistent and stage dependent.	Qualitative detection is feasible; negatives have limited exclusion value and post-treatment positives are ambiguous.	Portable/one-pot formats reduce transfer; extraction, specimen adequacy, and contamination controls remain important.	No intended-use evidence identified for added clinical utility beyond serology or faster treatment.	Narrow lesion-associated adjunct claim; insufficient evidence for universal screening or congenital exclusion.
Hypothetical universal single-tube maternal-infant triplex	No direct intended-use evidence for a single maternal-infant triplex claim.	Required specimens, analytes, and testing times rarely coincide.	Outputs differ: qualitative infant NAT, calibrated HBV quantity, and a serology-led syphilis pathway.	Multiplexing adds biochemical, calibration, contamination, and interpretation complexity.	No operational or clinical-utility evidence identified for a universal triplex service model.	No coherent single-tube claim supported; shared service infrastructure with separately validated assays is more consistent with the clinical pathways.

The five appraisal domains correspond to the framework in Section “1.1 Literature search and study selection.” Direct intended-use evidence requires alignment of population, analyte, specimen, operator/setting, output, comparator, and timing with the proposed claim; use of patient-derived specimens alone is insufficient. Clinical positions are qualitative and claim-bounded, not technology-readiness scores or regulatory, WHO-endorsed, or consensus classifications. Citations are representative; the complete core study inventory is provided in Supplementary Table 2. ART, antiretroviral therapy; EID, early infant diagnosis; HBV, hepatitis B virus; HIV, human immunodeficiency virus; NAT, nucleic acid testing.

## 5 Clinical positioning and a translational strategy for maternal–infant use

The following framework is intended to guide clinical positioning and technology development rather than to serve as a uniform regulatory or product-readiness checklist.

### 5.1 Positioning CRISPR within existing diagnostic pathways

Clinical positioning should be defined before assay optimization. Replacement, reflex, add-on, and triage claims require different comparators, outputs, and tolerances for error; readiness therefore belongs to a specific assay–specimen–decision combination rather than to a nuclease or platform name. A replacement claim requires performance comparable to the established pathway, whereas reflex, add-on, or triage tests must demonstrate incremental value without compromising safety or accuracy.

Relative to existing diagnostic pathways, the potential contribution of CRISPR is claim-specific. For HIV EID, the relevant benchmark is validated qualitative NAT, including established point-of-care molecular platforms; a CRISPR system would need to offer shorter total actionable turnaround, lower equipment or infrastructure burden, or easier decentralized deployment without compromising recovery from microvolume whole blood or dried blood spots, quality control, or diagnostic performance. For maternal HBV, the most plausible incremental role is same-visit DNA reflex testing after HBsAg positivity if specimen preparation and calibration can support IU/mL-linked classification without central-laboratory referral. For syphilis, established rapid serology already provides low-infrastructure same-visit screening, so direct CRISPR detection would need to add clinically useful information in selected lesion-associated, seronegative-window, or discordant cases rather than replace serology. Across all three applications, the identified CRISPR studies provide limited direct evidence on complete-pathway cost, intended-user quality control, equity, or linkage to clinical action; these potential advantages therefore remain to be demonstrated prospectively.

Because the three priority applications occur at different times and require different analytes, specimens, and result formats, service integration is more coherent than a universal reaction-level triplex. A shared reader, quality system, and workflow can support separately validated pathogen-specific assays while preserving distinct clinical claims. The pathogen-specific evidence supporting these positions is summarized in Section “4 Pathogen-specific evidence and translational readiness” and Table 3.

### 5.2 Determinants of translational success

Translation can be operationalized as four claim-dependent gates: intended-specimen recovery; analytical robustness of the locked test system; decision validity against the appropriate comparator; and operational or clinical utility within the intended service pathway. Detailed analytical and control requirements are described in Section “3 Design requirements for maternal and infant CRISPR testing” and Supplementary Table 4, whereas application-specific validation targets are summarized in Table 2. Later-stage endpoints such as same-visit result return, time to clinical action, pathway cost, and equity should be evaluated only when the study makes operational or clinical-utility claims.

### 5.3 Evidence-informed development and validation specifications for priority clinical applications

Table 2 translates the evidence synthesis into author-proposed development and validation specifications for the three priority applications. It identifies measurable validation domains and study expectations rather than fixed numerical acceptance thresholds. These specifications are not regulatory, WHO-endorsed, or consensus target product profiles and complement diagnostic-accuracy reporting guidance, including STARD 2015 (Bossuyt et al., 2015). They also serve a different purpose from Supplementary Table 4: specifies what complete-system studies should report, whereas Table 2 specifies what future application-specific validation should demonstrate. Table 2 distinguishes externally derived clinical or methodological requirements from author-proposed validation specifications using source-basis tags; where a cell combines more than one basis, the relevant tags are shown together.

The specifications preserve the distinct clinical endpoints identified earlier: intended-specimen qualitative EID with robust low-positive and invalid-result handling; IU/mL-traceable maternal HBV boundary classification; and specimen- and stage-specific syphilis add-on detection with explicit non-exclusion language for negative results. Detailed acceptance criteria should be prespecified for the target population, comparator pathway, and consequences of error.

### 5.4 A staged pathway from analytical validation to clinical impact

Development should proceed through four escalating stages. Stage 1 is analytical characterization of a locked configuration in the intended matrix, including specimen-level recovery, probabilistic detection limits where relevant, precision, inclusivity, interference, calibration or threshold behavior, contamination control, stability, lot variability, and invalid-result criteria. Results should be reported in units and effective volumes appropriate to the clinical claim; Supplementary Table 4 lists the corresponding reporting items.

Stage 2 is prospective evaluation in the intended-use population. Index-test thresholds and algorithms should be prespecified, operators blinded to the reference result where appropriate, and discordant or indeterminate results managed by a defined adjudication plan. Diagnostic accuracy, agreement, invalid and repeat rates, and other claim-specific performance measures should be reported separately from implementation outcomes.

Stage 3 is multisite operational evaluation by intended users in representative settings. Total turnaround time, hands-on time, environmental and operator failures, reader downtime, reagent wastage, result transmission, and external quality-assurance procedures should be assessed under realistic conditions.

Stage 4 asks whether the test improves care and does so equitably. Pragmatic designs should evaluate timely result receipt and clinical action, missed or unnecessary interventions, retention or follow-up where relevant, safety consequences of erroneous results, and pathway-level costs. Scale-up or multiplexed deployment should follow only after earlier stages demonstrate intended-specimen recovery, reproducibility, valid error handling, and clinical performance in the intended-use pathway.

## 6 Limitations and evidence gaps

Study-specific limitations are reported in Section “4 Pathogen-specific evidence and translational readiness” and Supplementary Tables 2, 3; the present section addresses limitations of the review process and of the evidence base as a whole. This review has methodological limitations. It was designed as a structured narrative clinical-pathway review rather than a prospectively registered systematic review. PubMed/MEDLINE and Europe PMC were the primary bibliographic databases, supplemented by citation chaining and targeted metadata verification; relevant engineering studies outside these routes may therefore have been missed. Because the review was not prospectively managed as a systematic review, screening counts and a PRISMA flow diagram were not retrospectively reconstructed. These constraints limit claims of exhaustive coverage even though the search was updated through 18 August 2026.

The evidence itself is highly heterogeneous in analyte, specimen matrix, amplification chemistry, readout, reporting unit, comparator, and clinical design, precluding meaningful meta-analysis across most applications. Pregnancy-specific, neonatal, and infant data remain sparse, so inferences about prophylaxis-exposed infants, HBV classification near maternal thresholds, and congenital syphilis rely partly on adjacent populations or analytical architectures. QUADAS-2 was applied only to patient-specimen diagnostic-accuracy studies for which the tool was appropriate; proof-of-concept and engineering studies were instead characterized by evidence stage and intended-use match. Preprints and mechanism-focused studies can inform design but cannot establish clinical readiness.

Operational and economic conclusions remain largely inferential because few studies compare CRISPR with the complete care pathway or measure patient-level outcomes. Within the studies identified by this review, we did not identify a complete product-level package covering intended-specimen recovery, locked thresholds, integrated controls, multisite reproducibility, and clinical impact. The positions in Table 3 are therefore qualitative, evidence-informed judgments across predefined domains rather than formal technology-readiness scores or regulatory, WHO, or consensus classifications; they should be revised as prospective intended-use and implementation evidence emerges.

## 7 Conclusion

Across the studies identified in this review, the principal translational constraints were intended-specimen integration, calibrated interpretation, error control, and intended-use validation. We did not identify a complete product-level evidence package sufficient for routine triple-elimination deployment. The near-term priority is therefore prospective validation of narrowly defined assay–specimen–decision combinations, with complete-system performance and time to clinical action assessed against established pathways.

Clinical priority and evidentiary maturity are not the same. HIV EID represents the most compelling near-term development opportunity in this synthesis; maternal HBV has the broadest clinical-specimen evidence but still lacks IU/mL-traceable pregnancy boundary validation; and syphilis supports only a narrow, specimen- and stage-specific adjunctive claim. Taken together, the evidence favors shared readers and quality systems with separately validated pathogen-specific assays over a universal single-tube triplex test.
