# Supplementary material for: CRISPR diagnostics for maternal–infant HIV, hepatitis B, and syphilis care: clinical roles and translational gaps
Source: Front Microbiol. 2026 Oct 21;17:1977927. doi: 10.3389/fmicb.2026.1977927 (PMC13732308; doi:10.3389/fmicb.2026.1977927)
Supplement: Supplementary file 1 [file Data_Sheet_1.PDF]

Supplementary Material

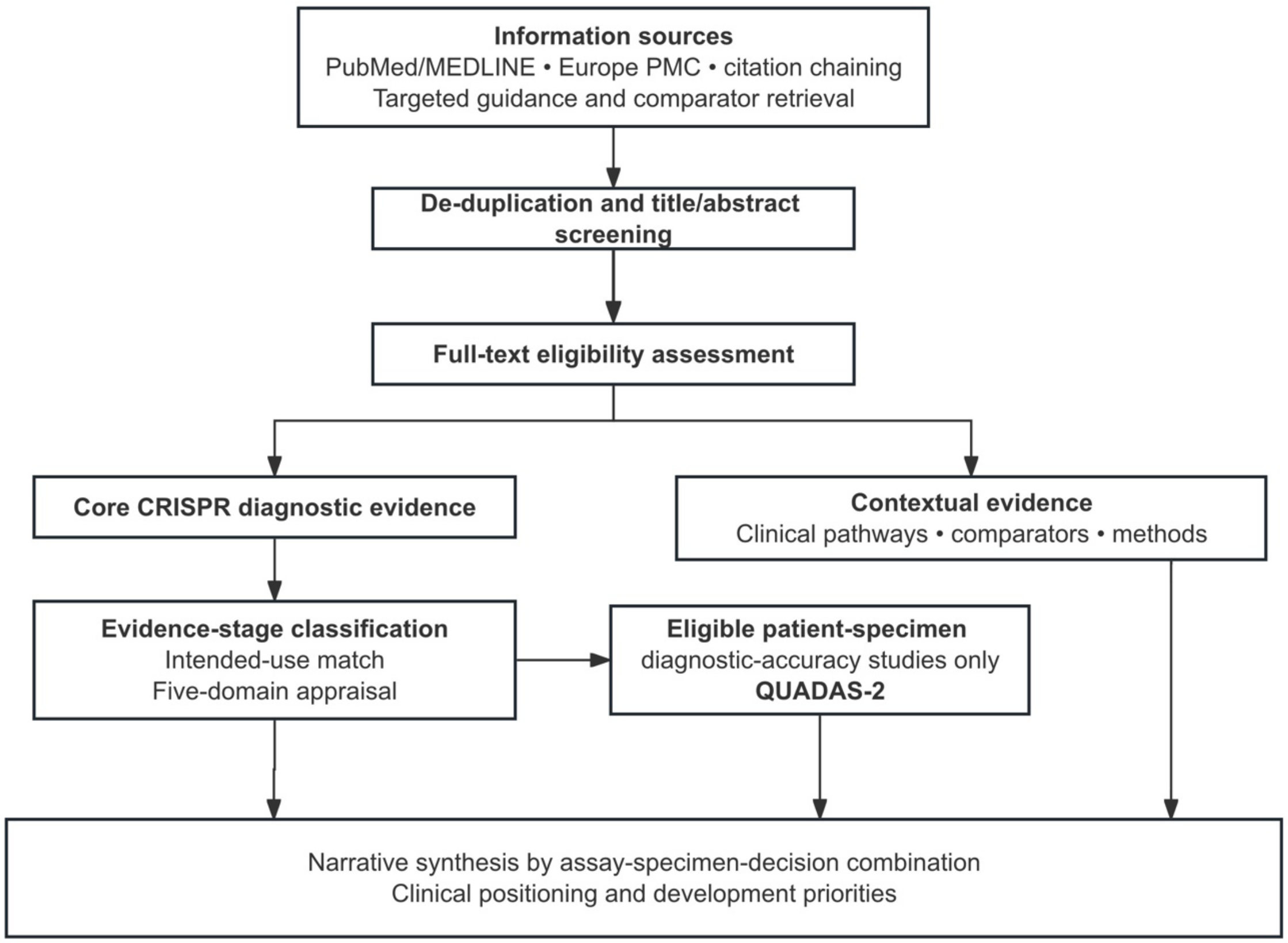

Supplementary Figure S1. Descriptive workflow for literature identification, study selection, evidence-stage classification, intended-use appraisal, and selective QUADAS-2 assessment. QUADAS-2 was applied only to eligible patient-specimen diagnostic-accuracy studies. Numerical screening counts are not shown because they were not prospectively recorded and were not reconstructed retrospectively.

Supplementary Tables

Supplementary Table S1. Literature search strategy, eligibility criteria, de-duplication, screening, and study selection process.

Component	Detailed search / selection specification	Operational rule / application
A. Review scope and information sources	A. Review scope and information sources	A. Review scope and information sources
Review design	Structured narrative, clinical-pathway review. The core evidence set comprised original CRISPR diagnostic studies for HIV, HBV, and *T. pallidum*. Contextual evidence defined maternal–infant clinical pathways, comparator tests, implementation constraints, and general engineering failure modes. A structured narrative design was selected because the review question concerns clinical positioning across heterogeneous assay architectures, specimens, outputs, and evidence stages rather than estimation of a single pooled diagnostic effect.	The review was not labelled as a systematic review; contextual evidence was not used to establish pathogen-specific clinical readiness, and no pooled readiness score was used. Structured searching and explicit eligibility, extraction, and appraisal procedures were used for transparency without implying systematic-review exhaustiveness.
Search period / last update	Database inception to 18 August 2026, inclusive. The final update search and reference audit were performed on 18 August 2026.	No lower date limit. Records published online ahead of print by the cut-off date were eligible.
PubMed/MEDLINE	Primary bibliographic source for peer-reviewed biomedical literature. Searches used Title/Abstract terms for CRISPR/Cas, each pathogen, and diagnostic concepts; targeted maternal/infant and comparator searches were run separately.	Core discovery source; DOI/PMID were captured where available.
Europe PMC	Complementary life-science search using TITLE_ABS terms and FIRST_PDATE ending 2026-08-18; used to capture PubMed-indexed records, open full text, and preprints relevant to emerging platforms.	Preprints were treated as provisional and superseded by the peer-reviewed article when a matched final publication existed.
Google Scholar / citation chaining	Used for targeted supplementary discovery, exact-title searches, "cited by" forward chasing, and backward reference-list screening from key primary studies and reviews.	Used as a supplementary discovery route rather than as the sole source of the core evidence set.
Crossref and publisher metadata	Exact title/DOI queries were used to verify bibliographic metadata, publication status, journal version, and DOI identity.	Verification source, not a substitute for eligibility screening.
WHO and guideline sources	WHO publication pages and relevant clinical guidance were searched for triple elimination, HBV prevention/diagnosis, syphilis screening/treatment, and maternal–infant pathways; guideline references were used as contextual evidence rather than CRISPR assay evidence.	Guidance was selected for clinical positioning and comparator pathways.
Databases not retrospectively claimed	Embase, Scopus, and Web of Science are not listed as searched because an auditable export/search log from those databases was not available for this narrative review.	This avoids retrospective overstatement of systematic-review coverage.
B. Search concepts and representative search strings	B. Search concepts and representative search strings	B. Search concepts and representative search strings
CRISPR/Cas concept block	(CRISPR OR "clustered regularly interspaced short palindromic repeats" OR "CRISPR-Cas" OR Cas9 OR Cas12* OR Cas13* OR Cas14* OR SHERLOCK OR DETECTR OR CARMEN OR HOLMES)	Used broadly so that platform names and nuclease variants were not missed.
HIV concept block	(HIV OR "human immunodeficiency virus" OR "HIV-1" OR "HIV-2" OR AIDS)	Core pathogen block; HIV-1/HIV-2 terms were retained even though the diagnostic literature was predominantly HIV-1.

Component	Detailed search / selection specification	Operational rule / application
HBV concept block	(HBV OR "hepatitis B" OR "hepatitis B virus" OR HBsAg OR "HBV DNA" OR cccDNA OR pgRNA)	Broad enough to identify direct DNA detection, quantitative/threshold concepts, and biomarker-development studies.
Syphilis concept block	(syphilis OR "Treponema pallidum" OR "T. pallidum")	Used for direct-detection and genotyping studies; clinical-stage and specimen terms were added in targeted searches.
Diagnostic concept block	(diagnos* OR detect* OR assay* OR biosensor* OR test* OR screening OR "point-of-care" OR "point of care" OR POC OR quantif* OR "viral load" OR genotyp* OR multiplex*)	Captures qualitative detection, quantitative/threshold outputs, genotyping, and near-patient formats.
Maternal–infant context block	(pregnan* OR antenatal OR prenatal OR perinatal OR maternal OR "mother-to-child" OR "vertical transmission" OR infant* OR neonat* OR "early infant diagnosis" OR EID OR congenital)	Run as a targeted supplementary block, not as a mandatory condition on every CRISPR search, because many relevant engineering/clinical assay studies were conducted in adjacent populations.
PubMed example – HIV	((CRISPR[Title/Abstract] OR "CRISPR-Cas"[Title/Abstract] OR Cas9[Title/Abstract] OR Cas12*[Title/Abstract] OR Cas13*[Title/Abstract] OR SHERLOCK[Title/Abstract] OR DETECTR[Title/Abstract] OR CARMEN[Title/Abstract] OR HOLMES[Title/Abstract]) AND (HIV[Title/Abstract] OR "HIV-1"[Title/Abstract] OR "HIV-2"[Title/Abstract] OR "human immunodeficiency virus"[Title/Abstract]) AND (diagnos*[Title/Abstract] OR detect*[Title/Abstract] OR assay*[Title/Abstract] OR biosensor*[Title/Abstract] OR "point-of-care"[Title/Abstract] OR quantif*[Title/Abstract] OR "viral load"[Title/Abstract] OR multiplex*[Title/Abstract]))	Date limit applied separately: database inception through 2026-08-18.
PubMed example – HBV	((CRISPR[Title/Abstract] OR "CRISPR-Cas"[Title/Abstract] OR Cas9[Title/Abstract] OR Cas12*[Title/Abstract] OR Cas13*[Title/Abstract] OR HOLMES[Title/Abstract]) AND (HBV[Title/Abstract] OR "hepatitis B"[Title/Abstract] OR "hepatitis B virus"[Title/Abstract] OR "HBV DNA"[Title/Abstract] OR cccDNA[Title/Abstract] OR pgRNA[Title/Abstract]) AND (diagnos*[Title/Abstract] OR detect*[Title/Abstract] OR assay*[Title/Abstract] OR biosensor*[Title/Abstract] OR quantif*[Title/Abstract] OR "viral load"[Title/Abstract] OR genotyp*[Title/Abstract] OR "point-of-care"[Title/Abstract]))	Date limit applied separately: database inception through 2026-08-18.
PubMed example – syphilis	((CRISPR[Title/Abstract] OR "CRISPR-Cas"[Title/Abstract] OR Cas12*[Title/Abstract] OR Cas13*[Title/Abstract]) AND (syphilis[Title/Abstract] OR "Treponema pallidum"[Title/Abstract] OR "T. pallidum"[Title/Abstract]) AND (diagnos*[Title/Abstract] OR detect*[Title/Abstract] OR assay*[Title/Abstract] OR biosensor*[Title/Abstract] OR "point-of-care"[Title/Abstract] OR multiplex*[Title/Abstract] OR genotyp*[Title/Abstract]))	Date limit applied separately: database inception through 2026-08-18.
Europe PMC adaptation	The same Boolean concept blocks were translated to TITLE_ABS: searches. Publication date was restricted with FIRST_PDATE to records first published on or before 2026-08-18. Preprint filters were reviewed separately so journal-published versions could be preferred.	Exact syntax may vary slightly by platform; the concept logic was preserved.
Targeted pathway searches	Pathogen + maternal–infant context terms were combined with comparator concepts such as NAT, viral load, HBsAg, HBeAg, serology, dried blood spot, early infant diagnosis, prophylaxis, congenital syphilis, implementation, turnaround time, and linkage to care.	These records were contextual and were not required to contain CRISPR terms.
Language / document availability	No language filter was imposed at the electronic-query level. Full-text eligibility required sufficient methodological detail for reliable extraction and interpretation.	Non-English records were eligible when the study could be reliably assessed from accessible full text

Component	Detailed search / selection specification	Operational rule / application
		or a reliable translation; records that could not be reliably assessed were not used to support readiness claims.
C. Eligibility criteria	C. Eligibility criteria	C. Eligibility criteria
Core inclusion criteria	Original primary research evaluating CRISPR/Cas-based detection, quantification, threshold classification, genotyping, multiplexing, or integrated diagnostic workflows for HIV, HBV, or T. pallidum. Clinical-specimen studies were prioritised; synthetic-target or spiked-matrix studies were retained only when they directly informed a predefined translational domain.	Eligible translational domains: specimen recovery, effective analysed volume, target inclusivity, calibration/traceability, internal controls, contamination containment, readout/interpretation, multiplexing, or sample-to-answer integration.
Contextual inclusion criteria	WHO/clinical guidance, reference-method studies, comparator NAT/serology evidence, maternal–infant implementation studies, and methodologic standards were included when needed to define the clinical decision, acceptable comparator, specimen, timing, or consequence of error.	Contextual studies were not treated as evidence of CRISPR clinical readiness.
Exclusion criteria	CRISPR gene-editing/therapy studies without a diagnostic output; unrelated pathogens without a direct platform-design rationale; reviews/editorials/letters without original assay data in the core evidence set; purely computational guide design without assay validation; patents/product brochures; conference abstracts lacking sufficient methods; duplicate reports; animal-only studies without direct diagnostic translation; and records with insufficient technical detail to classify the assay–specimen–decision combination.	Reviews could seed citation chasing but did not substitute for primary-study appraisal.
Preprints	Eligible only when uniquely informative and clearly identified as provisional. When a peer-reviewed journal article matched the same study, the final article superseded the preprint.	Preprints could inform design but were not used to establish clinical readiness.
Borderline records	If title/abstract information was insufficient to decide eligibility, the record advanced to full-text review rather than being excluded early.	Conservative rule to reduce false exclusion.
D. De-duplication, screening, and study selection	D. De-duplication, screening, and study selection	D. De-duplication, screening, and study selection
Record management	Search outputs and citation-chained records were consolidated into a master reference set with title, first author, year, journal, DOI, PMID/PMCID where available, and publication status.	A single study could have more than one report; reports were linked before study-level interpretation.
De-duplication – pass 1	Exact matching on DOI (case-insensitive; URL prefixes removed) and PMID/PMCID.	Exact identifier matches were collapsed automatically/manually to one record.
De-duplication – pass 2	Normalised title matching (lowercase, punctuation/extra whitespace removed) plus first author and publication year; near-matches were adjudicated manually.	Used for records with missing or malformed identifiers.
Preprint/final-publication de-duplication	Title, author list, methods, sample size, and DOI/journal linkage were compared. A journal article superseded the matched preprint; material unique to a preprint was not double-counted.	Prevents treating one study as two independent evidence items.
Stage 1 screening	Title/abstract screening removed obvious gene-editing therapy, unrelated disease, non-diagnostic, non-original, or technically irrelevant records.	Borderline records advanced to Stage 2.
Stage 2 screening	Full text was assessed for pathogen, intended analyte, specimen, original/effective input volume, assay architecture, comparator, controls, output, clinical population, and relevance to a maternal–infant decision or translational domain.	The study was then classified as direct intended-use evidence, indirect/platform-informing evidence, or contextual evidence.

Component	Detailed search / selection specification	Operational rule / application
Study selection hierarchy	Priority was given to: (1) prospective intended-use clinical evidence; (2) retrospective clinical-specimen evidence; (3) spiked intended-matrix feasibility; and (4) synthetic/mechanistic proof-of-concept. Lower-stage studies were retained when they added a distinct engineering or failure-mode insight. Core pathogen-specific studies used in the readiness synthesis are enumerated in Supplementary Table S2.	The evidence hierarchy prevents lower-stage engineering performance from being treated as equivalent to clinical validation.
Study-selection accounting	A numerical PRISMA-style flow diagram was not reconstructed because screening counts were not prospectively recorded. Instead, a non-numerical descriptive workflow is provided in Supplementary Figure S1 to distinguish study selection, evidence-stage appraisal, intended-use appraisal, and selective QUADAS-2 assessment.	No screening counts were fabricated post hoc. Supplementary Figure S1 is descriptive rather than a reconstructed PRISMA diagram.
E. Data extraction, appraisal, and synthesis	E. Data extraction, appraisal, and synthesis	E. Data extraction, appraisal, and synthesis
Data extraction fields	Pathogen/application; intended clinical decision; analyte and target locus; specimen/matrix and original volume; pre-analytical processing; Cas nuclease; amplification; readout; sample size; comparator/reference method; analytical LOD; clinical sensitivity/specificity/agreement; calibration units; genotype/subtype coverage; controls; open/closed workflow; hands-on/total time; invalid/indeterminate handling; and maternal–infant relevance.	These fields align with Supplementary Tables S2, S4, and S5 and the complete-system appraisal in the manuscript.
Evidence stage	Each study was classified as analytical proof-of-concept, spiked-matrix feasibility, retrospective clinical-specimen evaluation, prospective intended-use evaluation, or implementation evidence.	Engineering feasibility was not treated as equivalent to clinical validation.
Intended-use match	Appraisal considered whether the evaluated population, analyte, specimen, operator, setting, output, comparator, and timing matched the proposed maternal–infant claim.	Clinical readiness belongs to an assay–specimen–decision combination, not to the nuclease alone.
Structured narrative appraisal	Evidence was appraised across five predefined domains: direct intended-use evidence; integration of the intended specimen; validity of measurement/interpretation; maturity of controls/containment; and operational or clinical-utility evidence. The narrowest defensible position was based on the least mature domain and the consequence of error.	No numerical or ordinal readiness score was assigned.
Qualitative domain-maturity rubric	Direct intended-use evidence: synthetic/spiked or adjacent-population evidence → prospective intended-use population. Specimen integration: purified/extracted target → intended specimen with a validated front end. Measurement/interpretation: uncalibrated signal or broad-range association → prespecified qualitative/threshold rule or traceable quantitative agreement. Controls/containment: reaction-only controls or open workflow → specimen-adequacy, full-process, and readout controls within a contained workflow. Operational/clinical utility: laboratory feasibility → intended-user, multisite operational performance and demonstrated linkage to clinical action.	This rubric is directional rather than numeric. No domain score was assigned; the least mature domain bounded the evidence-informed clinical position.
Quantitative synthesis	Meta-analysis was not performed because studies differed markedly in analyte, specimen, sample preparation, amplification, readout, reporting unit, comparator, and clinical spectrum; reaction-level LODs were not converted across incomparable effective volumes.	Raw denominators, exact confidence intervals, invalid/indeterminate results, and discordant adjudication were prioritised when available.
Formal risk-of-bias tool	QUADAS-2 was applied to patient-specimen diagnostic-accuracy studies that compared an index CRISPR assay with a reference standard and for which the tool was applicable. Risk of bias was judged for patient selection, index test, reference standard, and flow/timing; applicability was	Judgements are reported in Supplementary Table S3 and are conservative: “unclear” was used when

Component	Detailed search / selection specification	Operational rule / application
	judged for patient selection, index test, and reference standard. Proof-of-concept, spiked-matrix, platform-engineering, and primarily quantitative-method-comparison studies were not forced into QUADAS-2 and were characterised by evidence stage and intended-use match instead.	reporting did not permit a defensible low- or high-risk judgement.
Reference-integrity audit	Author–date citations, DOI identity, publication status, and duplicate references were re-audited after accepting tracked changes. WHO publication years were corrected to the publication year rather than the 2026 access year, and new studies identified in the final 18 August 2026 update were added to the evidence inventory.	Author-date citation correspondence and bibliographic metadata were cross-checked against the final cited records; matched peer-reviewed publications superseded preprints where applicable.

Supplementary Table S2. Core pathogen-specific CRISPR diagnostic studies that directly informed the translational-readiness synthesis.

Pathogen / application	Study (ref.)	Architecture / output	Material and sample size	Key result reported in manuscript	Main translational limitation	Maternal–infant relevance	Evidence stage; intended-use relevance
HIV / multiplex surveillance	Ackerman et al. (2020)	CARMEN–Cas13; pre-amplification, droplet pairing, microscopy, computational multiplex readout	11,268 interpretable sample–assay result pairs from a smaller number of clinical specimens	11,236/11,268 pairwise agreement with sequencing; resistance agreement 86–90%, increasing to 93% after excluding highly mismatched targets	Complex reference-laboratory workflow; pairwise results are not independent patient tests	Surveillance/sequence-inclusivity insight rather than near-patient maternal–infant testing	Retrospective clinical-specimen evaluation; indirect surveillance use
HIV / clinical RNA detection	Li et al. (2023)	RT-RAA–Cas13a with fluorescence/lateral-flow readout	158 clinical plasma samples	Sensitivity 91.81%, specificity 100%; lowest reported detectable viral load 112 copies/mL	Plasma/extraction workflow; not infant-specific; no microvolume whole-blood/DBS EID validation	Important clinical detection evidence, but indirect for EID	Clinical-specimen diagnostic-accuracy study; indirect for EID
HIV / one-pot clinical detection	Uno et al. (2023)	RT-RPA separated from CRISPR-Cas12a by agarose gel in one vessel	14 clinical plasma samples plus analytical targets	Clinical plasma results were compared with real-time RT-PCR; assay demonstrated one-pot clinical feasibility	Small clinical set; extracted plasma RNA; one-hour reaction and imaging requirements	Containment concept relevant to EID system engineering, not direct EID validation	Small clinical-specimen study; platform-informing
HIV / signal amplification	Moon et al. (2025)	CARRD anti-tag hairpin feedback with Cas13a fluorescence	30 HIV plasma specimens plus analytical targets	AUC 0.9709 for group discrimination; analytical LOD 10 aM for HIV/HCV RNA	Extraction required; limited clinical sample size; hairpin leakage/manufacturing stability unresolved	Mechanistic signal amplification, not a validated EID or viral-load claim	Small clinical-specimen plus analytical study; platform-informing
HIV / digital viral load	Nouri et al. (2023)	Amplification-free membrane digital Cas13a (STAMP)	20 patient plasma specimens plus spiked plasma/synthetic RNA	Reported sensitivity 60%, specificity 100%; estimated specimen-level detection limit ~2,000 copies/mL	Only ~50 nL of extracted RNA entered membrane; low-load performance limited by effective volume	Demonstrates digital quantification but not replacement of maternal viral-load testing	Small clinical-specimen plus spiked-matrix study; indirect for maternal viral load
HIV / digital threshold	Ngo et al. (2024)	RT-RPA–Cas12a digital microwell counting with smartphone/deep learning	Synthetic targets	Detected 75 RNA copies/reaction; early readout produced fewer positive wells than endpoint	No patient validation; algorithm/device configuration requires locking	Potential component for portable quantitative or threshold testing	Analytical synthetic-target study; platform-informing
HIV / threshold classification	Song et al. (2025)	dCas9 magnetic micromotor velocity (dCRISTOR)	9 patient plasma specimens	9/9 classified correctly relative to a 1,000 copies/mL threshold	Very small clinical series; threshold output rather than	Exploratory urgent-triage concept	Very small clinical-specimen

Pathogen / application	Study (ref.)	Architecture / output	Material and sample size	Key result reported in manuscript	Main translational limitation	Maternal–infant relevance	Evidence stage; intended-use relevance
					traceable quantification		study; indirect threshold concept
HIV / EID	Gaur et al. (2026)	Single-tube RPA–Cas12a targeting clade C pol; fluorescence	41 seropositive plasma specimens, including 28 children <2 years, plus healthy-donor controls	Infant subset 27/28 positive initial results and one inconclusive; full evaluation reported 96% sensitivity and 92.65% specificity	Plasma RNA extraction/reverse transcription; no DBS or microvolume whole-blood validation; clade-C-bounded claim	Most directly relevant current EID study, but not a deployable replacement	Infant-focused clinical-specimen study; direct clinical question with specimen mismatch
HIV / syphilis duplex	Xu et al. (2026)	One-pot RT, multiplex RPA, transcription, Cas12a/Cas13a, dual-line lateral flow	46 clinical samples plus analytical targets	HIV sensitivity 100%, specificity 97.5%; T. pallidum sensitivity 90%, specificity 100% vs qPCR	Small mixed panel; different clinical pathways; extraction-free lysis and line-elimination readout need intended-use validation	Demonstrates duplex chemistry, not a universal maternal–infant claim	Small clinical evaluation; indirect platform-oriented match
HBV / clinical detection and resistance	Wang et al. (2021)	PCR–T7–Cas13a fluorescence and mutation genotyping	280 serum specimens; additional resistance cohort	PPA 100% (90/90), NPA 94.7% (180/190) vs qPCR; discordant low-copy signals incompletely adjudicated	Open multistage workflow; additional CRISPR-positive calls not fully adjudicated	Supports detection, not maternal reflex threshold classification	Retrospective clinical-specimen study; indirect for maternal reflex use
HBV / low-level viraemia	Tian et al. (2023)	RAA–T7–Cas13a lateral flow	180 participants with HBV infection; 111 longitudinal specimens from 23 treated patients	PPA ~87% in low-level viraemia and 100% above 100 IU/mL; longitudinal PPA 100%, NPA 92.15%	Four CRISPR-positive/qPCR-negative results not fully adjudicated; limited quantitative traceability	Shows specimen-level constraints below 100 IU/mL	Clinical-specimen/longitudinal study; indirect for maternal threshold use
HBV / digital measurement	Cai et al. (2025)	Digitised RPA and Cas12a in paired/electrofused droplets (DIMERIC)	24 clinical serum specimens plus dilution series	R^2 0.92033 vs qPCR and 0.97337 vs ddPCR; slightly lower concentration estimates	No Bland–Altman limits or boundary-focused replication; 20-min reaction excluded front-end/analysis	Technical basis for threshold classification, not interchangeable clinical viral load	Small clinical-specimen plus dilution study; indirect threshold concept
HBV / one-tube detection	Xu et al. (2024)	LAMP–AapCas12b at 60 °C (HOLMESv2)	236 clinical specimens	198/200 qPCR-positive and 36/36 qPCR-negative detected; genotype subgroup signal of mismatch-associated failure	Copy conversion assumed ideal recovery; no IU/mL traceability; genotype limitation signal	Strong clinical feasibility with unresolved calibration and inclusivity risks	Clinical-specimen study; indirect for maternal reflex use

Pathogen / application	Study (ref.)	Architecture / output	Material and sample size	Key result reported in manuscript	Main translational limitation	Maternal–infant relevance	Evidence stage; intended-use relevance
HBV / integrated whole-blood workflow	Zong et al. (2022)	Centrifugal microfluidics with separation, extraction, and genotype detection	Simulated rather than patient specimens	Integrated whole-blood separation and magnetic extraction in a closed disc	No patient-specimen validation	Useful system-integration prototype for maternal near-patient testing	Simulated-matrix integration study; platform-informing
HBV / clinical Cas13a detection	Sata et al. (2026)	RPA/nested-RPA–T7–Cas13a kinetic fluorescence	45 human clinical samples in the HBV cohort (plus 30 HCV samples)	Assay differentiated HBV-positive and healthy samples in an Indian cohort	No pregnancy-specific threshold classification or IU/mL-traceable boundary validation	Adds clinical detection evidence but remains indirect for maternal reflex use	Clinical-specimen study; indirect for maternal threshold use
HBV / dual DNA/RNA clinical detection	Li et al. (2026c)	Dual PCR–Cas12a/Cas13a fluorescence (DPCFS)	46 serum samples from 22 treatment-naïve and 24 treated patients with chronic HBV infection	The authors reported HBV DNA sensitivity of 97.06% and specificity of 100% versus ultrasensitive qPCR; RNA sensitivity was 86.84% and specificity 87.5% versus RT-ddPCR	Designed for treatment monitoring; no pregnancy cohort or decision-boundary validation	Strengthens clinical detection evidence, not maternal prophylaxis classification	Clinical-specimen diagnostic-accuracy study; indirect for maternal reflex use
HBV / light-activated one-pot detection	Zhou et al. (2026)	Light-activated enzymatic recombinase amplification (ERA)–Cas12a; fluorescence and lateral flow in a sealed one-pot format	79 clinical serum samples	The authors reported 100% sensitivity by fluorescence and 96.3% by lateral flow; Cohen κ 0.94–1.00 versus qPCR; 30-min assay	No IU/mL traceability or pregnancy boundary study; UV activation remains a workflow requirement	Strong one-pot clinical feasibility but not maternal threshold validation	Clinical-specimen diagnostic-accuracy study; indirect for maternal reflex use
HBV / dual HDV–HBV detection	Tian et al. (2026)	RT-RAA–Cas13a/Cas12a dual fluorescence/lateral-flow system	70 plasma specimens from participants with HBV/hepatitis D virus (HDV) coinfection	Positive concordance was 85.7% (60/70) by fluorescence and 82.9% (58/70) by lateral flow	Co-infected population; extracted plasma; qualitative detection rather than traceable maternal classification	Multiplex engineering evidence, not a triple-elimination intended-use claim	Clinical-specimen study; indirect multiplex evidence
Syphilis / direct detection and genotyping	Chen et al. (2022)	PCR–T7–LwCas13a fluorescence	216 specimens from primary/secondary syphilis cases and controls	Mixed-matrix sensitivity 93.3%, specificity 100%; performance highest in lesions and lower in blood; lineage/resistance demonstrated	Aggregate estimate cannot be extrapolated to pregnancy, latent or congenital disease; open laboratory workflow	Benchmark for specimen-stratified direct detection	Clinical-specimen study; indirect for pregnancy or congenital use

Pathogen / application	Study (ref.)	Architecture / output	Material and sample size	Key result reported in manuscript	Main translational limitation	Maternal–infant relevance	Evidence stage; intended-use relevance
Syphilis / RPA–Cas12a clinical detection	Li et al. (2026b)	RPA–Cas12a fluorescence and lateral flow targeting tpp47/tp0574 (reported as tp47 in the source study)	30 clinically characterised specimens from suspected syphilis cases; the report describes peripheral blood collection and later refers to serum in the clinical validation	The authors reported 96.6% overall diagnostic accuracy, reflecting high concordance with the study's serology-based clinical diagnosis	Small cohort; automated extraction; blood/serum rather than preferred lesion/mucosal-swab initial claim	Useful clinical evidence for blood-based add-on testing; indirect for maternal/lesion intended use	Clinical diagnostic-accuracy study; indirect specimen match
Syphilis / portable multiplex	Low et al. (2026)	Portable dual-target CRISPR fluorescence	900 extracted-DNA specimens; 400 in T. pallidum–HSV arm	T. pallidum sensitivity 82.5%; 17/18 initially false-negative specimens positive on repeat	Retrospective reference-laboratory evaluation beginning with extracted DNA; repeat testing complicates same-visit use	Potential add-on platform, not universal antenatal screening	Retrospective extracted-DNA clinical study; indirect add-on use
Syphilis / one-pot direct detection	Wu et al. (2026)	Single-tube RPA–T7–Cas13a; fluorescence and lateral flow	186 specimens across four centres, including whole blood, lesion exudate, and CSF	Fluorescence analytical LOD 10 copies/µL; ~50-min total time; reported 100% specificity; lesion exudate highest sensitivity	Extraction remains; lateral-flow LOD ~200 copies/µL; low organism burden in blood/CSF remains limiting	Supports a narrow specimen-specific add-on claim	Multicentre clinical-specimen study; indirect, specimen-specific use

Supplementary Table S3. QUADAS-2 appraisal of patient-specimen diagnostic-accuracy studies used in the clinical-readiness synthesis.

Study / application	Risk of bias (four QUADAS-2 domains)	Applicability concerns (three QUADAS-2 domains)	Key rationale
Li et al. (2023), HIV RNA	Patient selection: Unclear; Index test: Unclear; Reference standard: Low; Flow/timing: Unclear.	Patient selection: High; Index test: High; Reference standard: Low.	Large clinical plasma series with RT-qPCR comparator, but recruitment and threshold-prespecification details are insufficient for consistently low risk-of-bias judgements. Adult/plasma workflow does not match microvolume infant EID.
Gaur et al. (2026), HIV EID	Patient selection: High; Index test: Unclear; Reference standard: Unclear; Flow/timing: Low.	Patient selection: High; Index test: High; Reference standard: Unclear.	Seropositive specimens plus healthy-donor controls create spectrum-selection concerns; some positive specimens lacked a measured viral-load comparator. The infant clinical question is direct, but the index workflow uses extracted serum/plasma and a clade-C-bounded assay rather than intended microvolume whole blood/DBS.
Wang et al. (2021), HBV	Patient selection: High; Index test: Unclear; Reference standard: Low; Flow/timing: Unclear.	Patient selection: High; Index test: High; Reference standard: Low.	A qPCR-positive/qPCR-negative clinical set is vulnerable to spectrum-selection effects, and discordant low-copy results were incompletely adjudicated. The index test was not designed for pregnancy decision-boundary classification.
Tian et al. (2023), HBV low-level viraemia	Patient selection: Unclear; Index test: Unclear; Reference standard: Low; Flow/timing: Low.	Patient selection: High; Index test: High; Reference standard: Low.	A clinically defined low-level-viraemia cohort and longitudinal specimens improve spectrum relevance, but index-test threshold prespecification is incompletely reported for QUADAS-2 purposes. Population and output do not match an HBsAg-positive pregnancy boundary cohort.
Xu et al. (2024), HBV HOLMESv2	Patient selection: High; Index test: Unclear; Reference standard: Low; Flow/timing: Low.	Patient selection: High; Index test: High; Reference standard: Low.	Evaluation used separated qPCR-positive and negative specimens and small genotype subgroups. It supports clinical detection but not IU/mL-traceable classification near a maternal prophylaxis boundary.
Sata et al. (2026), HBV	Patient selection: High; Index test: Unclear; Reference standard: Unclear; Flow/timing: Unclear.	Patient selection: High; Index test: High; Reference standard: Unclear.	Positive and healthy human samples support feasibility but create selection-spectrum concerns. Available reporting does not establish a maternal decision-boundary reference design or intended pregnancy population.

Study / application	Risk of bias (four QUADAS-2 domains)	Applicability concerns (three QUADAS-2 domains)	Key rationale
Li et al. (2026c), HBV DNA/RNA	Patient selection: Unclear; Index test: Unclear; Reference standard: Low; Flow/timing: Low.	Patient selection: High; Index test: High; Reference standard: Low.	Clinical serum comparison used ultrasensitive qPCR/RT-ddPCR comparators, but the intended purpose was chronic-HBV treatment monitoring. Neither the population nor index-test output matches maternal threshold classification.
Zhou et al. (2026), HBV one-pot	Patient selection: Unclear; Index test: Unclear; Reference standard: Low; Flow/timing: Unclear.	Patient selection: High; Index test: High; Reference standard: Low.	Clinical serum agreement with qPCR supports one-pot detection feasibility, but reporting available for the review does not permit low-risk judgements for all selection/threshold domains. No pregnancy boundary or IU/mL-traceability evaluation was performed.
Chen et al. (2022), syphilis	Patient selection: High; Index test: Unclear; Reference standard: Unclear; Flow/timing: Unclear.	Patient selection: High; Index test: High; Reference standard: High.	Primary/secondary syphilis cases and controls create spectrum concerns; mixed matrices and multiple reference approaches complicate a single reference-standard judgement. Pregnancy/congenital populations and intended high-yield maternal specimen claims were not evaluated.
Li et al. (2026b), syphilis	Patient selection: Unclear; Index test: Low; Reference standard: Unclear; Flow/timing: Low.	Patient selection: High; Index test: High; Reference standard: Low.	Thirty suspected cases were tested with operator blinding and a predefined fluorescence rule. The report describes peripheral blood collection and later refers to serum in the clinical validation; the serology-based clinical reference is appropriate for clinical diagnosis but imperfect for adjudicating direct pathogen detection.
Low et al. (2026), syphilis/STI panel	Patient selection: Unclear; Index test: Low; Reference standard: Low; Flow/timing: Low.	Patient selection: High; Index test: High; Reference standard: Low.	Masked retrospective extracted-DNA evaluation reduces index-test interpretation bias, but reference-laboratory specimens and repeat testing do not match same-visit maternal add-on use.
Wu et al. (2026), syphilis	Patient selection: Unclear; Index test: Unclear; Reference standard: Unclear; Flow/timing: Unclear.	Patient selection: High; Index test: High; Reference standard: Unclear.	Multicentre clinical specimens improve generalisability, but matrix heterogeneity and incomplete reporting of selection/reference procedures limit risk-of-bias certainty. No dedicated pregnancy or congenital cohort was evaluated.

Study / application	Risk of bias (four QUADAS-2 domains)	Applicability concerns (three QUADAS-2 domains)	Key rationale
Xu et al. (2026), HIV–syphilis duplex	Patient selection: High; Index test: Unclear; Reference standard: Low; Flow/timing: Low.	Patient selection: High; Index test: High; Reference standard: Low.	The small mixed clinical panel with qPCR comparator supports duplex chemistry but is vulnerable to spectrum effects and does not represent a coherent maternal–infant dual-target intended-use population.

Abbreviations used in Supplementary Tables S2–S3: AUC, area under the receiver operating characteristic curve; CHB, chronic hepatitis B; CSF, cerebrospinal fluid; DBS, dried blood spot; ddPCR, droplet digital PCR; EID, early infant diagnosis; ERA, enzymatic recombinase amplification; HDV, hepatitis D virus; LFA, lateral-flow assay; LOD, limit of detection; NPA, negative percent agreement; PPA, positive percent agreement; qPCR, quantitative PCR; RAA, recombinase-aided amplification; RPA, recombinase polymerase amplification; RT-ddPCR, reverse-transcription droplet digital PCR; RT-RAA, reverse-transcription recombinase-aided amplification.

Supplementary Table S4. Claim- and stage-dependent complete-system reporting checklist for CRISPR diagnostic studies.

Domain	Minimum information to report	Applicability by stage/claim and rationale	Pathogen-specific emphasis
Intended use and clinical position	Replacement, reflex, add-on, triage, surveillance, or research use; clinical action and comparator pathway	Core for all studies: state the intended analytical, engineering, or clinical claim. Performance requirements depend on the assigned role and consequence of error.	EID replacement vs maternal HIV triage; HBV prophylaxis classification; syphilis add-on detection
Population and spectrum	Recruitment method, inclusion/exclusion criteria, age, pregnancy status, disease stage, treatment/prophylaxis exposure, and prevalence	Required for clinical-specimen or intended-use claims; analytical/model-system studies should instead define the material tested. Spectrum-restricted case–control panels can inflate accuracy.	Infant specimens with low target concentrations; HBsAg-positive pregnant women near boundary; specimen/stage-stratified syphilis
Specimen definition	Matrix, collection device, original volume, storage, transport, freeze–thaw history, processing time, and adequacy criteria	Required whenever performance is claimed for a biological or intended-use specimen. Clinical performance is inseparable from specimen type and handling.	Microvolume blood/DBS; serum/plasma/whole blood; lesion, blood, placenta, tissue, or CSF
Pre-analytical recovery	Lysis, extraction/direct release, concentration, elution volume, reaction input, recovery estimate, and effective analysed volume	Required for intended-specimen or sample-to-answer claims; not mandatory for purified-target mechanism studies. Reaction-level LOD cannot be converted to specimen-level performance without these data.	Critical for low-copy HIV and blood-based syphilis; required for HBV IU/mL traceability
Target and inclusivity	Target locus, primer and guide sequences or accessions, in silico databases and search date, variant panels, genotype/subtype distribution, and call logic for multi-target assays	Core for target-specific claims, with validation breadth matched to the claimed population. Mismatches in primers or guides can cause sequence-dependent failure.	HIV-1 diversity; HBV genotypes and overlapping reading frames; conserved *T. pallidum* targets

Domain	Minimum information to report	Applicability by stage/claim and rationale	Pathogen-specific emphasis
Controls	Specimen-adequacy, full-process, amplification/inhibition, CRISPR/readout, negative, contamination, and high-load/dilution controls with acceptance criteria	Reaction-level controls are expected for analytical studies; specimen-adequacy and full-process controls apply to intended-specimen clinical claims. Controls distinguish true negatives from collection, extraction, inhibition, reagent, or readout failure.	Human genomic control for cellular blood; alternative adequacy control for plasma; matrix-specific tissue controls
Assay architecture and containment	Amplification chemistry, temperatures, timings, concurrent/sequential steps, manual triggers, open transfers, cartridge seals, waste containment, and carryover monitoring	Core reporting item; containment expectations increase for near-patient or decentralised claims. "One-pot" and portable readout do not necessarily mean closed or sample-to-answer operation.	Open-transfer risk is particularly important for high-amplification near-patient workflows
Analytical performance	Replicate design, 95% detection probability, precision, linearity, reportable range, interference, cross-reactivity, stability, lot variation, carryover, and invalid criteria	Applicable to analytical-performance claims. Single best-case LODs do not establish reproducibility.	HBV high-load saturation and boundary precision; HIV low-copy detection; syphilis matrix interference
Quantification or threshold classification	Measurand, units, calibrators, traceability chain, dilution rules, grey zone, allowable bias/error, uncertainty, and out-of-range rules	Applicable only to quantitative, semi-quantitative, or clinical-threshold claims. Correlation across a broad range does not establish agreement or boundary classification.	Essential for HBV; required for maternal HIV viral load; usually not appropriate for syphilis direct detection
Clinical accuracy	Raw 2 × 2 counts, confidence intervals, reference standard, blinding, invalids, repeats, indeterminates, threshold derivation, and discordant adjudication	Applicable to patient-specimen diagnostic-accuracy claims. Denominators and result handling directly affect reported performance.	Report low-positive strata and prophylaxis; genotype strata; specimen/stage-specific syphilis
Software and reader	Hardware, optics, phone model, algorithm/software version, locked threshold, training/validation datasets, cybersecurity, and update control	Applicable when algorithmic or instrument-assisted interpretation forms part of the test system. Software may not transfer across devices or lots without validation.	Important for digital counting, imaging, and automated classification

Domain	Minimum information to report	Applicability by stage/claim and rationale	Pathogen-specific emphasis
Operational performance	Hands-on and total time, initial invalid and repeat rates, operator errors, environmental failures, downtime, reagent wastage, maintenance, connectivity, and result transmission	Applicable to intended-user, near-patient, or implementation claims. A rapid reaction may still produce a slow or fragile service workflow.	Same-visit result and linkage to ART/prophylaxis/treatment
Clinical utility and harm	Result receipt, time to action, retention, missed interventions, unnecessary treatment, false reassurance, cost of the complete pathway, and equity outcomes	Applicable only to clinical-utility or implementation claims. Diagnostic value is realised when a valid result changes care without unintended harm.	False-negative HIV EID; HBV threshold error; negative syphilis direct test
Evidence stage and validation design	Analytical proof-of-concept, spiked-matrix, retrospective clinical-specimen, prospective intended-use, or implementation study; recruitment, prespecification, blinding, invalid/indeterminate handling, and discordance plan	Core for all studies: state the evidence stage and validation design. Different stages support different claims; analytical or engineering proof-of-concept is not penalised for lacking later-stage clinical utility, but cannot by itself establish product readiness.	Direct infant pathway for EID; HBsAg-positive pregnant women near the HBV decision boundary; stage- and matrix-stratified syphilis cohorts

Note: This checklist is claim- and stage-dependent and should not be interpreted as requiring early analytical or engineering studies to demonstrate later-stage clinical accuracy, implementation performance, or clinical utility.

Supplementary Table S5. Standardised terminology used for CRISPR diagnostic development and clinical positioning.

Term	Definition used in this review	Common source of overstatement or confusion
Analytical LOD	Target concentration or amount associated with a prespecified detection probability under defined analytical conditions	A single positive dilution or copies/reaction value is presented as a clinical LOD
Clinical detection performance	Sensitivity or agreement observed in intended specimens and populations using the complete workflow	Synthetic or spiked-matrix results are treated as clinical accuracy
Effective analysed volume	The portion of the original specimen whose target molecules can contribute to the final readout	Nominal collection volume is assumed to equal the volume interrogated by the assay
Qualitative detection	Classification of target as detected or not detected, with invalid and indeterminate categories	A qualitative signal is relabelled as viral load or used as an exclusion result without validation
Threshold classification	Classification relative to a clinical action point, including a prespecified uncertainty/grey zone	High overall correlation is used instead of estimating error near the boundary
Metrologically traceable quantification	A continuous result linked through an unbroken calibration hierarchy to a stated reference, with uncertainty and reportable range	Fluorescence intensity or plasmid copies are treated as interchangeable with IU/mL
Correlation	Association between two measurements across a range	High R^2 is interpreted as agreement or interchangeability
Agreement	Closeness of measurements or classifications, assessed using bias, limits of agreement, or boundary-specific error	Agreement is reported without clinically relevant error limits
One-pot	Amplification and CRISPR steps occur in one physical vessel, whether simultaneously or sequentially	Assumed to mean closed, simultaneous, extraction-free, or fully automated
Closed system	Post-amplification material remains contained during routine operation and readout	A lateral-flow or second-tube transfer is described as closed because the core reaction is sealed
Sample-to-answer	The system begins with the intended clinical specimen and integrates preparation, detection, interpretation, controls, and waste containment	A reaction starting with purified nucleic acid is described as sample-to-answer
Near-patient testing	Testing located closer to care than a central reference laboratory; it may still require trained staff or a hub	Used interchangeably with point-of-care or same-visit testing without operational evidence
Point-of-care testing	Testing performed at or near the site of care by intended users with an actionable result in the clinical workflow	Portability of the reader is treated as sufficient evidence of point-of-care readiness
Same-visit result	A valid result is returned before the patient leaves or before the relevant decision must be made	Core reaction time is reported without total workflow and result-transmission time
Reaction-level multiplexing	Multiple targets are detected in the same biochemical mixture	Technical multiplexing is assumed to create a coherent multi-pathogen clinical claim
Cartridge-level multiplexing	Physically separated reaction chambers share one disposable and reader	Separate assays are described as a single chemistry despite different conditions and calibration
Service-platform integration	Instruments, training, procurement, quality assurance, connectivity, and data systems are shared while assays retain separate claims	Service integration is conflated with a universal triplex reaction

Term	Definition used in this review	Common source of overstatement or confusion
Specimen-adequacy control	A control that provides evidence that suitable biological material was collected	An amplification control is assumed to verify collection adequacy
Full-process control	A control that passes through preparation, amplification, CRISPR detection, and readout	A no-template or reaction-only control is treated as sufficient for patient-level validity
Invalid result	The assay fails predefined control or quality criteria, so no clinical interpretation is permitted	Invalids are excluded from accuracy denominators without disclosure
Indeterminate result	The assay functions but the signal lies in a predefined uncertainty zone requiring repeat or confirmatory testing	Indeterminate results are silently counted as negative or removed
Evidence stage	The level of evidence generated by the study: analytical proof-of-concept, spiked-matrix feasibility, retrospective clinical-specimen evaluation, prospective intended-use evaluation, or implementation evidence	Engineering feasibility is treated as equivalent to intended-use clinical validation
Intended-use match	The degree to which the evaluated population, analyte, specimen, operator, setting, output, and comparator match the proposed clinical claim	Use of patient-derived material is labelled as direct evidence even when the specimen or clinical decision differs from the proposed use
Bounded clinical position	A deliberately narrow evidence-informed statement defining the population, specimen, output, and role currently supported, without implying regulatory or guideline endorsement	A platform capability or broad pathogen-detection result is generalised to universal screening, replacement, or exclusion claims

References

Ackerman, C. M., Myhrvold, C., Thakku, S. G., Freije, C. A., Metsky, H. C., Yang, D. K., et al. (2020). Massively multiplexed nucleic acid detection with Cas13. Nature 582, 277–282. doi: 10.1038/s41586-020-2279-8

Bossuyt, P. M., Reitsma, J. B., Bruns, D. E., Gatsonis, C. A., Glasziou, P. P., Irwig, L., et al. (2015). STARD 2015: an updated list of essential items for reporting diagnostic accuracy studies. BMJ 351, h5527. doi: 10.1136/bmj.h5527

Cai, D., Wang, Y., Zhang, Z., Huang, E., Yang, N., Yang, X., et al. (2025). Droplet pairing-merging enabled digital RPA-CRISPR/Cas12a (DIMERIC) assay for rapid and precise quantification of Hepatitis B Virus DNA. Biosens. Bioelectron. 276, 117256. doi: 10.1016/j.bios.2025.117256

Chen, W., Luo, H., Zeng, L., Pan, Y., Parr, J. B., Jiang, Y., et al. (2022). A suite of PCR-LwCas13a assays for detection and genotyping of Treponema pallidum in clinical samples. Nat. Commun. 13, 4671. doi: 10.1038/s41467-022-32250-y

Gaur, A., Bhakhri, H., Mishra, N., Sharma, S., Bansal, T., Kalaivani, M., et al. (2026). A rapid CRISPR/Cas12a-based assay for the detection of HIV-1 Clade C in infants. Clin. Chim. Acta 578, 120518. doi: 10.1016/j.cca.2025.120518

Li, W., Sun, Y., Ye, M., Liang, Y., Ouyang, J., Xu, W., et al. (2026b). Rapid visual detection of Treponema pallidum using the RPA-CRISPR/Cas12a system. Sci. Rep. 16, 5120. doi: 10.1038/s41598-026-35745-6

Li, X., Su, B., Yang, L., Kou, Z., Wu, H., Zhang, T., et al. (2023). Highly sensitive and rapid point-of-care testing for HIV-1 infection based on CRISPR-Cas13a system. BMC Infect. Dis. 23, 627. doi: 10.1186/s12879-023-08492-6

Li, Y., Liu, Y., Wu, L., Chen, Y., Wang, M., Luo, H., Chen, J., and Hu, Y. (2026c). Simultaneous and ultrasensitive detection of HBV DNA and RNA in CHB by utilizing Cas12a/Cas13a-based dual-target strategy. Sens. Biosensing Res. 51, 100925. doi: 10.1016/j.sbsr.2025.100925

Low, S. J., O'Neill, M. T., Fernando, J. A., Kerry, W. J., Prestedge, J., Wild, N., et al. (2026). CRISPR-Cas-based diagnostics for point-of-care detection of sexually transmitted infections: a laboratory development and evaluation study. Lancet Microbe 7, 101289. doi: 10.1016/j.lanmic.2025.101289

Moon, J., Zhang, J., Guan, X., Yang, R., Guo, C., Schalper, K. T., et al. (2025). CRISPR anti-tag-mediated room-temperature RNA detection using CRISPR/Cas13a. Nat. Commun. 16, 9142. doi: 10.1038/s41467-025-64205-4

Ngo, H. T., Akarapipad, P., Lee, P.-W., Park, J. S., Chen, F.-E., Trick, A. Y., et al. (2024). Rapid and portable quantification of HIV RNA via a smartphone-enabled digital CRISPR device and deep learning. Sens. Actuators Rep. 8, 100212. doi: 10.1016/j.snr.2024.100212

Nouri, R., Jiang, Y., Politza, A. J., Liu, T., Greene, W. H., Zhu, Y., et al. (2023). STAMP-Based Digital CRISPR-Cas13a for Amplification-Free Quantification of HIV-1 Plasma Viral Loads. ACS Nano 17, 10701–10712. doi: 10.1021/acsnano.3c01917

Sata, T. N., Sah, A. K., Ismail, M., Sharma, G., Doloi, R., Nayak, B., Shalimar, and Venugopal, S. K. (2026). Development of RPA and nested-RPA based CRISPR/Cas13a diagnostic platform for the identification of HBV DNA and HCV RNA in Indian patient cohort. Diagn. Microbiol. Infect. Dis. 116, 117514. doi: 10.1016/j.diagmicrobio.2026.117514

Song, Y., Park, H., Thirumalaraju, P., Kovilakath, N., Hardie, J. M., Bigdeli, A., et al. (2025). Deactivated Cas9-Engineered Magnetic Micromotors toward a Point-of-Care Digital Viral RNA Assay. ACS Nano 19, 8646–8660. doi: 10.1021/acsnano.4c14913

Tian, Y., Cao, Y., Pan, Z., Xu, L., Fan, Z., Mo, Y., et al. (2026). CRISPR-Cas13a/Cas12a Assisted Dual Portable and Visualized HDV and HBV Detection. J. Med. Virol. 98, e70976. doi: 10.1002/jmv.70976

Tian, Y., Fan, Z., Xu, L., Cao, Y., Chen, S., Pan, Z., et al. (2023). CRISPR/Cas13a-assisted rapid and portable HBV DNA detection for low-level viremia patients. Emerg. Microbes Infect. 12, e2177088. doi: 10.1080/22221751.2023.2177088

Uno, N., Li, Z., Avery, L., Sfeir, M. M., and Liu, C. (2023). CRISPR gel: A one-pot biosensing platform for rapid and sensitive detection of HIV viral RNA. Anal. Chim. Acta 1262, 341258. doi: 10.1016/j.aca.2023.341258

Wang, S., Li, H., Kou, Z., Ren, F., Jin, Y., Yang, L., et al. (2021). Highly sensitive and specific detection of hepatitis B virus DNA and drug resistance mutations utilizing the PCR-based CRISPR-Cas13a system. Clin. Microbiol. Infect. 27, 443–450. doi: 10.1016/j.cmi.2020.04.018

Whiting, P. F., Rutjes, A. W. S., Westwood, M. E., Mallett, S., Deeks, J. J., Reitsma, J. B., et al. (2011). QUADAS-2: a revised tool for the quality assessment of diagnostic accuracy studies. Ann. Intern. Med. 155, 529–536. doi: 10.7326/0003-4819-155-8-201110180-00009

Wu, Q., Du, F., Zhang, X., Lu, Z., Zheng, X., Li, A., et al. (2026). Development and evaluation of a one-pot CRISPR/Cas13-based assay for syphilis detection across multiple clinical sample types. Int. J. Infect. Dis. 164, 108414. doi: 10.1016/j.ijid.2026.108414

Xu, H., Lin, G., Chen, R., Cai, Z., Sun, Y., Zhang, X., et al. (2024). CRISPR/Cas12b assisted loop-mediated isothermal amplification for easy, rapid and sensitive quantification of chronic HBV DNA in one-pot. Anal. Chim. Acta 1310, 342702. doi: 10.1016/j.aca.2024.342702

Xu, Z., Wei, M., Jiang, M., Wang, Y., and He, K. (2026). Single-step duplex CRISPR coupled with lateral flow assay for point-of-care detection of human immunodeficiency virus and Treponema pallidum. Anal. Methods 18, 1996–2004. doi: 10.1039/D5AY02168E

Zhou, X., Wang, L., Wei, C., Liu, M., Li, Y., Gui, F., Zhou, L., and Deng, Z. (2026). A light-activated one-pot ERA/CRISPR-Cas12a assay for cost-effective dual-mode detection of HBV DNA. Talanta, 130329. doi: 10.1016/j.talanta.2026.130329

Zong, N., Gao, Y., Chen, Y., Luo, X., and Jiang, X. (2022). Automated Centrifugal Microfluidic Chip Integrating Pretreatment and Molecular Diagnosis for Hepatitis B Virus Genotyping from Whole Blood. Anal. Chem. 94, 5196–5203. doi: 10.1021/acs.analchem.2c00337
